# Genomic and In Vivo Characterization of Antimicrobial Resistance and Virulence in UPEC *Escherichia coli* Isolated from Brazilian Cases of UTI

**DOI:** 10.1021/acsomega.5c06488

**Published:** 2025-11-12

**Authors:** Barbara Gatti Cardoso, Rodrigo Dias de Oliveira Carvalho, Bertram Brenig, Vasco Azevedo, Marisa Salvi, Geovanni Dantas Cassali, Simone Odilia Antunes Fernandes, Valbert Nascimento Cardoso

**Affiliations:** † Departamento de Análises Clínicas e Toxicológicas, Faculdade de Farmácia, Laboratório de Radioisótopos, 28114 Universidade Federal de Minas Gerais, Belo Horizonte, MG 31270-901, Brazil; ‡ Department of Molecular Biology, I Institute of Veterinary Medicine, Göttingen 37018, Germany; § Departamento de Genética, Ecologia e Evolução, Instituto de Ciências Biológicas, Laboratório de Biologia Celular e Molecular, 28114 Universidade Federal de Minas Gerais, Belo Horizonte, MG 31270-901, Brazil; ∥ Departamento de Patologia geral, Instituto de Ciências Biológicas, 28114 Universidade Federal de Minas Gerais, Belo Horizonte, MG 31270-901, Brazil

## Abstract

With the growing global prevalence of antimicrobial resistance, the treatment of urinary tract infections caused by *Escherichia coli* is becoming increasingly challenging. Uropathogenic *E. coli* (UPEC) is responsible for approximately 80% of urinary infections worldwide and possesses extraordinary genetic plasticity that facilitates both antimicrobial resistance and virulence. This study aimed to characterize the genomic profiles of four clinical UPEC strains (SJR07, SJR30, SJR31, and SJR49) that were recently isolated from women with UTIs in the city of Divinópolis, Brazil. Through whole-genome sequencing and comparative analysis, we identified a high degree of genetic diversity, including a heterogeneous repertoire of resistance and virulence genes located on plasmids and genomic islands. In particular, the extended spectrum beta-lactamase (ESBL) gene SHV-12 variant was found in a mobilizable plasmid in the genome of the SJR49 strain. Notably, phylogenetic and sequence-type (ST) analyses revealed the presence of globally relevant lineages such as ST10 and ST354, which are known for their adaptability and resistance potential. Functional assays using an experimental renal infection model demonstrated the ability of SJR49 to persist in the renal tissue and induce more severe functional and histopathological kidney damage. These findings highlight the urgent need to develop therapeutic strategies for community-acquired UPEC infections and mitigate the spread of multidrug-resistant strains.

## Graphical abstract

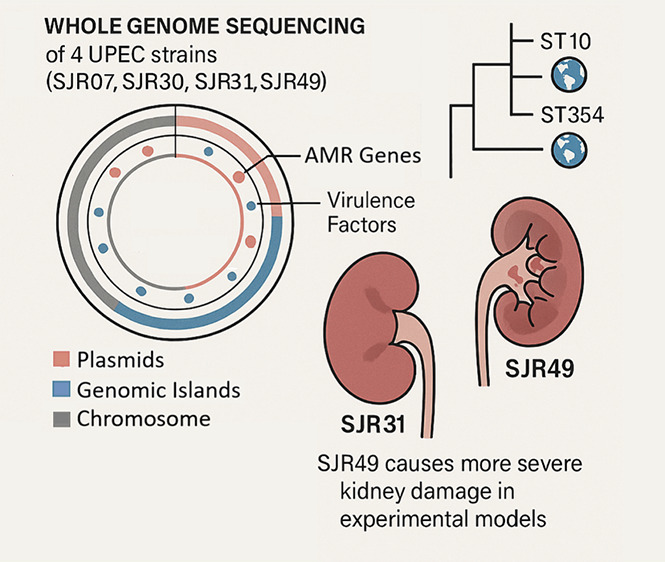

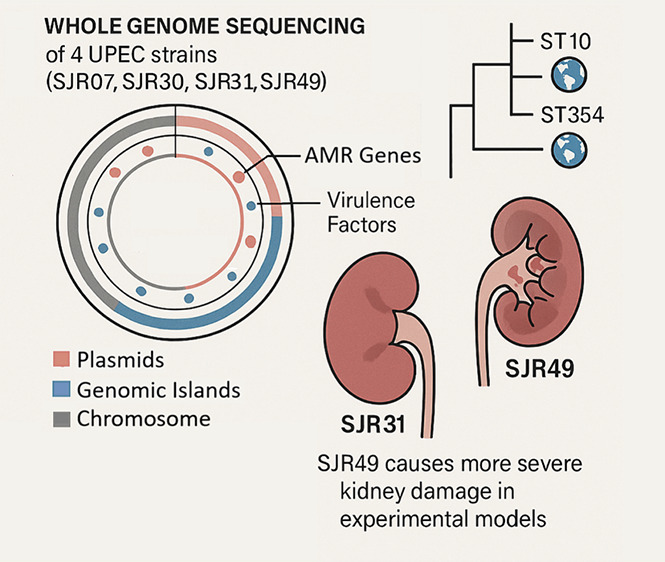

## Introduction

As the antimicrobial resistance crisis worsens, common diseases such as urinary tract infections (UTIs) are becoming increasingly difficult to treat.^1^ Among the major pathogens responsible for resistance-associated deaths, *E. coli* stands out as the main culprit. More than 20% of *E. coli* strains, the predominant pathogens in UTI, have demonstrated resistance to both the initially recommended drugs and secondary treatment options.^2^

*E. coli* has extraordinary genetic plasticity that allows it to adapt to different environments and hosts. As a result of this characteristic, *E. coli* can show different pathotypes with specific clinical behaviors, including uropathogenic *E. coli* (UPEC).^3^ Genetically diverse strains of UPEC are equipped with fitness determinants that allow them to migrate across the intestines. After leaving the gastrointestinal tract, UPEC adheres to the urethra and ascends into the bladder, causing cystitis. During bladder infection, these bacteria can reach the kidneys, causing pyelonephritis, and, in more severe cases, transfer to the bloodstream, resulting in bacteremia.^3^

UPEC is the causative agent of ∼80% of the 150 million cases of UTI recorded annually worldwide.^4^ Therefore, a thorough understanding of the factors that contribute to the antimicrobial resistance of these strains is essential. Mechanisms such as the production of extended-spectrum β-lactamases (ESBLs), the presence of efflux pumps, and the acquisition of resistance genes through mobile genetic elements confer *E. coli* a remarkable ability to persist in the face of selective pressure imposed by antibiotic use.^5^ Moreover, the virulence factors of UPEC, such as adhesins and siderophores, further complicate the clinical management of infections.^5^

The ability of *E. coli* to develop resistance and disseminate resistance genes reinforces the need for studies aimed at identifying local resistance patterns and characterizing the underlying genetic mechanisms. The genomic heterogeneity of UPEC is also extensive. Changes in genomic content can influence strain-specific phenotypes. Therefore, molecular profiling of clinical UPEC pathogens using whole-genome sequencing is critical for understanding the sequence heterogeneity in resistance and virulence alleles.^5^ Understanding these factors is crucial for developing new therapeutic and preventive strategies.

To address these issues, the present study sought to conduct a detailed analysis of antimicrobial resistance and virulence in clinical strains of UPEC (SJR49, SJR31, SJR030, and SJR07) recovered from the urine of women with clinical and laboratory diagnoses of catheter-associated (CA)-UTI by comparing them with global strains, as an essential component in combating UTIs. In vivo studies were performed using the radiopharmaceutical dimercaptosuccinic acid radiolabeled with technetium-99m (^99m^Tc-DMSA) to evaluate the renal parenchyma. This evaluation aimed to support the discussion of cases of impairment of the kidney’s functional unit (nephron) caused by infection. Imaging results and target-to-nontarget ratios below 1 indicate that ^99m^Tc-DMSA uptake by the kidneys was reduced.^6^

## Results

### Genome Quality Analysis and Assembly

All genomes of the samples were analyzed for completeness, contamination, total size, number of contigs, and GC content (Table S1). All samples showed 100% completeness, except for EC03, indicating complete genomes. Only strain P6175 presented a contamination rate above 5%, whereas the majority of contamination values were lower than 1%, reinforcing the quality of the genomic data. Genome sizes ranged from 4,302,255 to 5,453,963 bp, which were compatible with those expected for *E. coli* genomes. The GC content of the samples was approximately 50–51%, as expected for this species.

### Strain Allele-Typing Analysis and Taxonomic Analysis

All the strains showed average nucleotide identity (ANI) values above 96% with the reference genome *E. coli* strain K-12 GCF_000005845.2. In multilocus sequence typing (MLST) analysis, strains SJR07 and SJR49 belonged to sequence types (STs) 10 and ST167, respectively, and were classified as clonal complex (CC) 10. Both shared the same alleles, except for a difference in the *purA* allele (SJR07: allele 8; SJR49: allele 13), indicating a close genetic relationship between the two strains. In contrast, strain SJR30 was identified as belonging to ST448 Cplx with the sequence type ST448. This strain presented a distinct allele profile for all genes, indicating a distant genetic relationship with other strains. Strain SJR31 was classified as 354 Cplx and presented ST354, which contains alleles that were highly divergent in relation to the other strains analyzed (Table 1).

**Table 1:** MLST Classification of the Four Clinical Isolates of Resistant UPEC Strains^a^

			alleles						
strain	ST	clonal complex	*adk*	*fumC*	*gyrB*	*icd*	*mdh*	*purA*	*recA*
SJR07	10	ST10 Cplx	10	11	4	8	8	8	2
SJR30	448	448 Cplx	6	6	5	16	11	8	7
SJR31	354	354 Cplx	85	88	78	29	59	58	62
SJR49	167	ST10 Cplx	10	11	4	8	8	13	2

a The table presents strain identification based on sequence type (ST), the corresponding clonal complex (Clonal complex), and the alleles of seven loci from multilocus sequence typing (MLST): *adk* (adenylate kinase), *fumC* (fumarate hydratase C), *gyrB* (DNA gyrase subunit B), *icd* (isocitrate dehydrogenase), *mdh* (malate dehydrogenase), *purA* (adenylosuccinate synthetase), and *recA* (recombinase A).

### Phylogenetic Analysis

*E. coli* phylogenetic analysis revealed that the isolates belonged to different phylogenetic groups, as shown in Figures 1 and S3. Strain SJR30 was classified within phylogroup B1, a group frequently associated with commensal and community-acquired strains, although it did not show close relatedness with previously reported global strains. In contrast, strain SJR31 clustered in phylogroup F, in close proximity to the global ST354 strain PDT001090596. Strains SJR07and SJR49 were assigned to phylogroup A and grouped within the same clade together with the international ST10 lineages, L1LB, and the ST167 strain PDT001089438 respectively.

**Figure 1:**
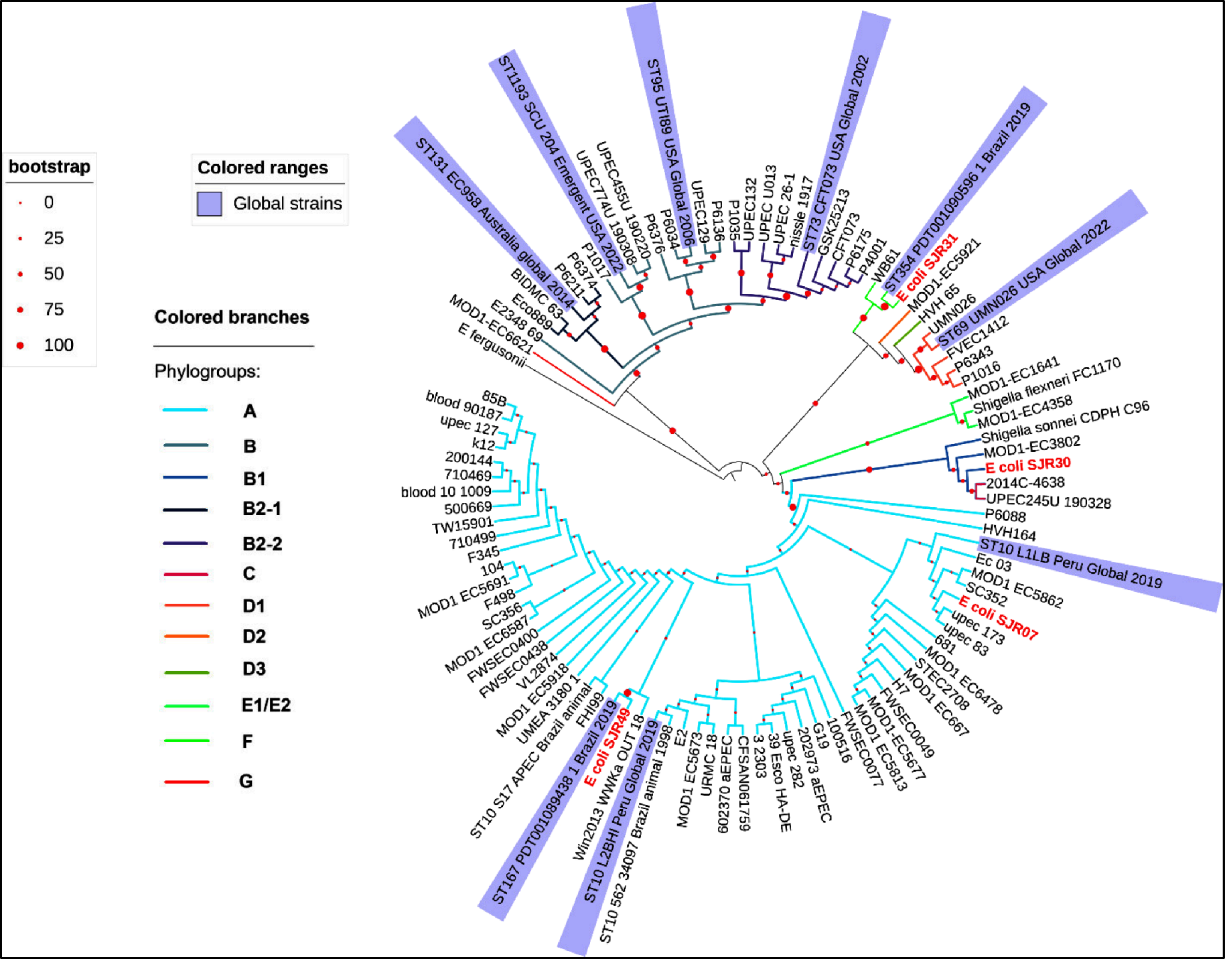
Multilocus phylogenetic tree based on representative genomes of different *Escherichia coli* phylogroups and globally disseminated clonal complex strains. Phylogenetic relationships among *E. coli* isolates based on the concatenated alignment of seven alleles (*adk, fumC, gyrB, icd, mdh, purA,* and *recA*; total length = 3.480 bp). The tree was inferred using the maximum-likelihood method under the GTRGAMMA evolutionary model with 1.000 bootstrap replicates. Bootstrap support values are indicated by red dots on branch bipartitions. Phylogroups are shown on the left panel, distinguished by different colors. The genome of *Escherichia fergusonii* was used as an outgroup.

### Genomic Analysis Revealed the Presence of Divergent Genes Related to Antimicrobial Resistance among Clinical Isolates

As identified by the Comprehensive Antibiotic Resistance Database (CARD), the determinants of antimicrobial resistance showed a heterogeneous profile among the genomes of the clinical isolates of *E. coli* UPEC (SJR07, SJR30, SJR31, and SJR49) analyzed in the present study and in relation to the other isolates reported in the literature (Figures 2 and S1).

**Figure 2:**
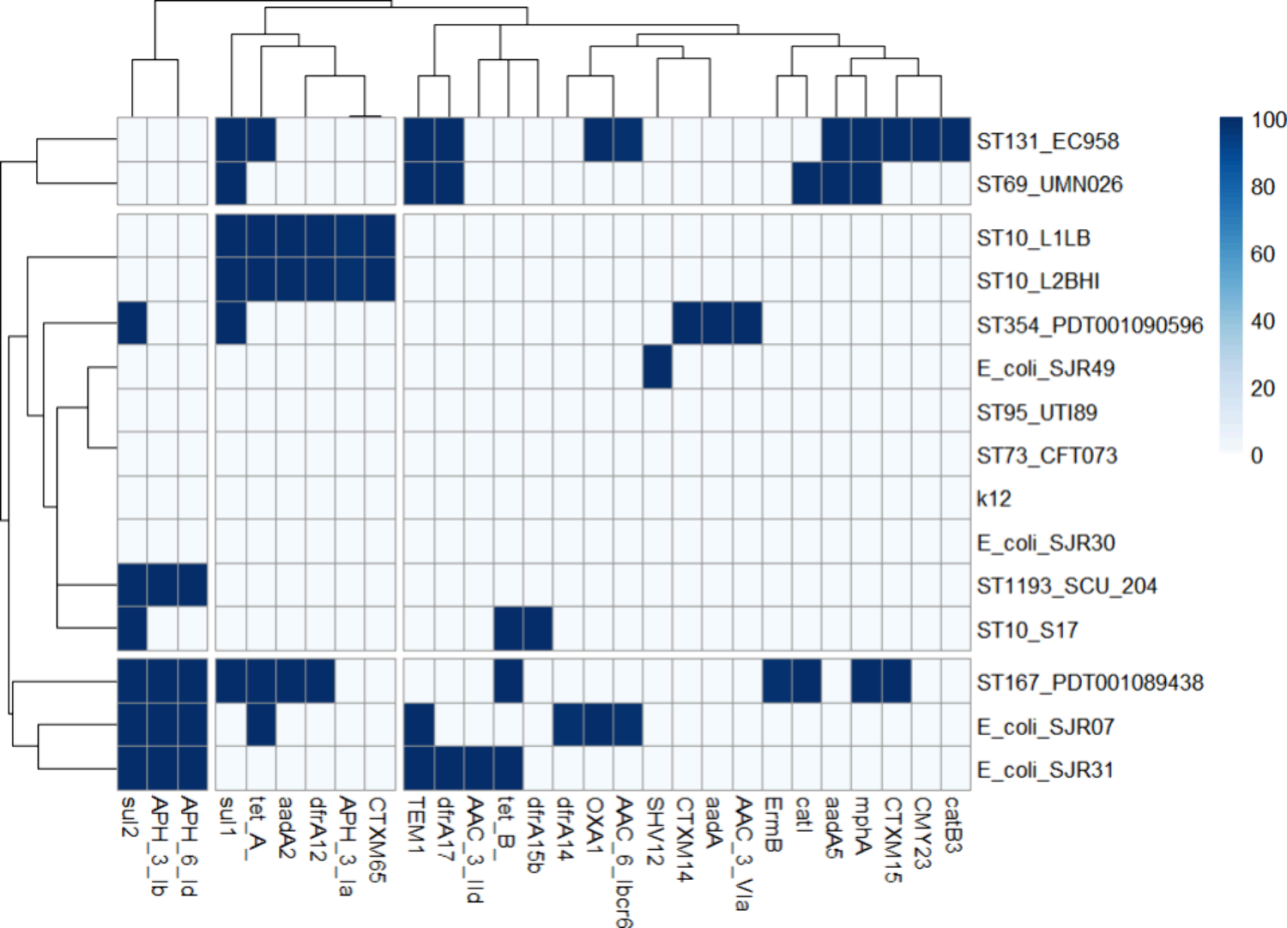
Antimicrobial resistance genes identified in the analyzed *Escherichia coli* genomes. Amino acid sequences from the strains isolated in this work and globally disseminated strains were aligned against the CARD database. Sequences showing at least 70% identity and 100% coverage were considered homologous. The presence or absence of resistance genes is represented in a heatmap, with each column corresponding to a gene and each row representing an *E. coli* strain.

Among the genes identified, those related to resistance to aminoglycosides stood out, namely, *aac­(3)-IId*; *AAC­(6′)-Ib-cr*, and *APH­(3”)-Ib*. Among these, *aac­(3)-IId* was identified in the SJR31 strain, and *AAC­(6′)-Ib-cr* was identified in strain SJR07. AAC(6′)-Ib was identified as the most clinically relevant acetyltransferase, being responsible for resistance to amikacin and other aminoglycosides in Gram-negative bacteria. Sulfonamide resistance was observed with the *Sul-2* gene in the SJR31 and SJR07 strains. None of the resistance genes identified in UPEC in the present study were found in *E. coli* K12, which was used as a control for nonpathogenic *E. coli*. On the other hand, the classic and emergent UPEC global strains (eg CFT073, PDT001090596, L1LB, and PDT001089438) presented several resistance genes.

### Genomic Analysis Revealed the Presence of Clinically Relevant Genes Related to Antimicrobial Resistance in Plasmids

Antimicrobial resistance genes were identified in the plasmids of clinical isolates using the CARD database, as shown in Figure 3 and Table S2. All analyzed strains contained plasmids: seven in SJR07, three in SJR49, two in SJR31, and one in SJR30. In addition, antimicrobial resistance genes were detected in the plasmids of the SJR49, SJR07, and SJR31 strains. The resistance genes *dfrA17*, *tet­(B)*, *sul2*, APH(3′)-IB, and APH(6′)-IB were identified in SJR07. OXA-1, *tet­(A)*, and AAC 6 IB CR6 genes were detected in SJR31. SHV-12 was detected only in the SRJ49 strain.

**Figure 3:**
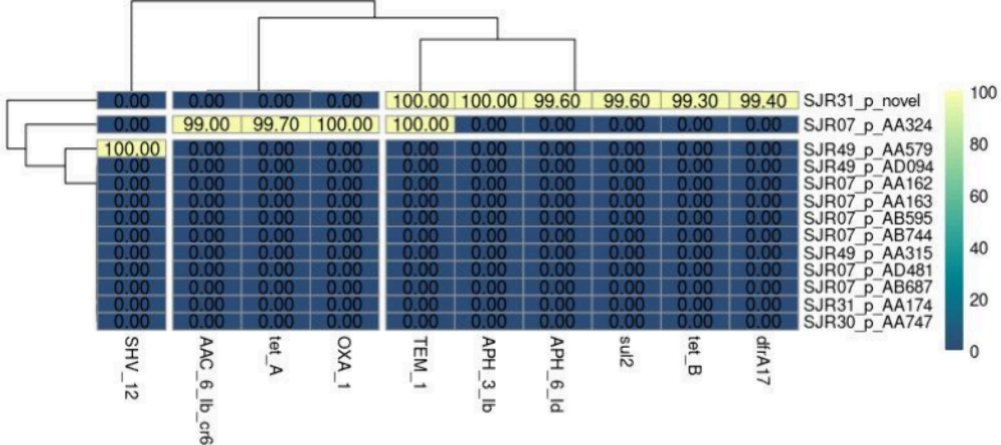
Antimicrobial resistance genes and virulence factors in plasmids from the strains isolated in this study. SHV-12 (class A beta-lactamase with extended-spectrum resistance to cephalosporin) AAC(6′)-IB-CR6 (acetyltransferase associated with aminoglycoside and fluoroquinolone resistance), *tetA* (efflux pump associated with tetracycline resistance), OXA-1 (class D β-lactamase), TEM-1 (class A β-lactamase), APH(3′)-B and APH(6)-ID (kinases related to aminoglycoside resistance), *sul2* (dihydropteroate synthase associated with sulfonamide resistance), *tetB* (efflux pump for tetracyclines), and *dfrA17* (dihydrofolate reductase associated with trimethoprim resistance).

### Genomic Analysis Revealed the Presence of Divergent Virulence-Related Genes among the Clinical Isolates

In the annotation performed using the Virulence Factor Database (VFDB), virulence determinants also showed a heterogeneous profile among the genomes of the clinical isolates of *E. coli* UPEC (SJR07, SJR30, SJR31, and SJR49) analyzed in the present study and in relation to the pathogenic isolates collected in the literature (Figures 4 and S2).

**Figure 4:**
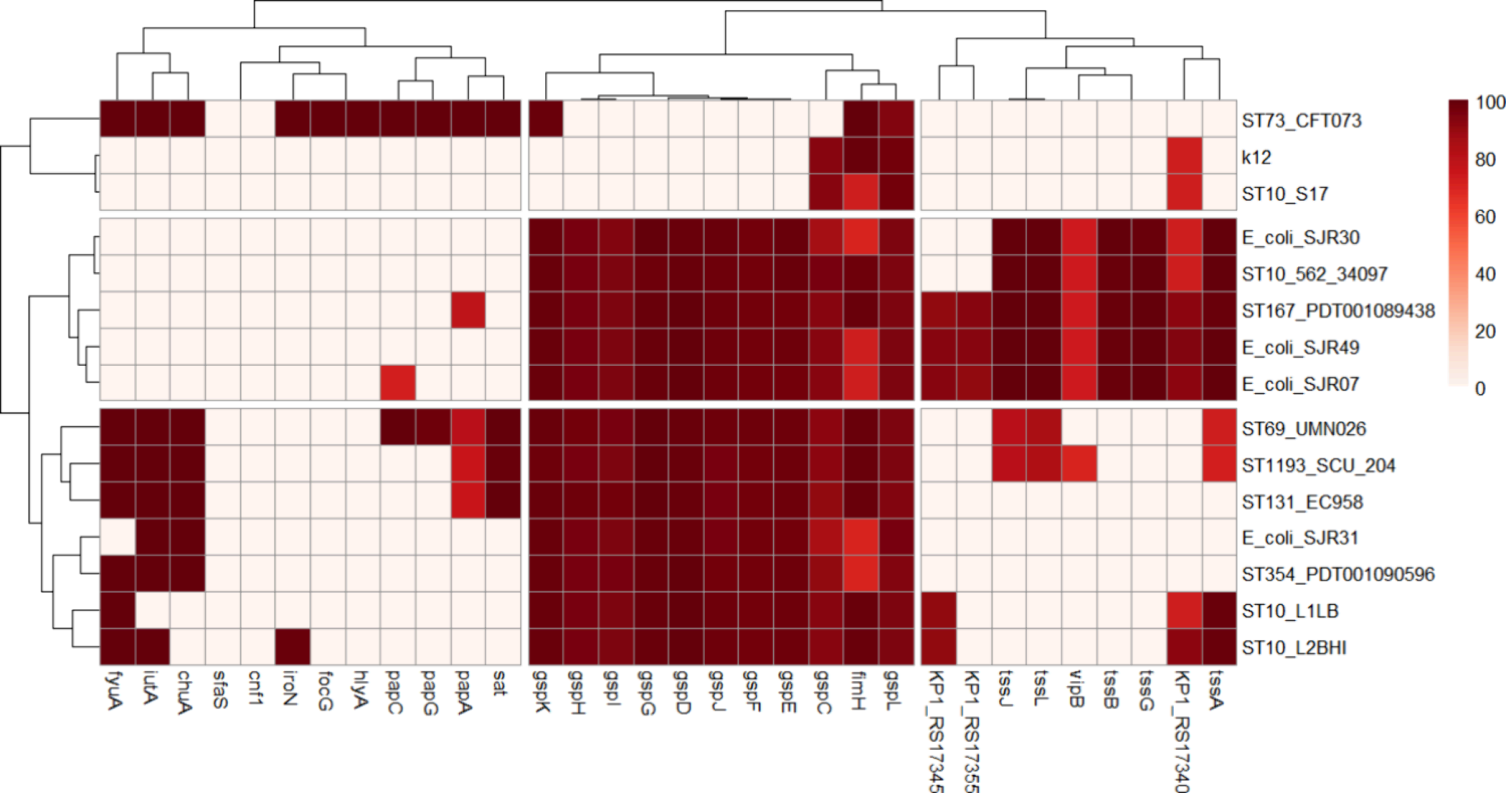
Virulence genes identified in the analyzed *Escherichia coli* genomes. Amino acid sequences from the strains isolated in this work and globally disseminated strains were aligned against the Virulence Factor Database (VFDB), considering as homologous those with at least 70% identity and 100% coverage. The presence or absence of virulence genes is shown in a heatmap, where columns represent genes and rows correspond to the different *E. coli* strains analyzed.

Among the prominent genes, the genes encoding pseudopilins (*gsp*), which are responsible for the secretion of proteins and virulence factors through the outer membrane of Gram-negative bacteria, were present in the four UPEC samples in the present study (SJR07, SJR30, SJR31, and SJR49), as well as in several global strains, but not in CFT073 strain. Aerobictin (*iuc*) and *chu* genes, which are used for iron acquisition by bacterial cells, and membrane channel component (KPS) genes were found in strains SJR31 and CFT073. The antiphagocytosis gene (KP1) and secretion system (*vipB* and *tss*) genes were also detected in strain SJR49.

### Genomic Islands Predicted the Presence of Several Genes Associated with Virulence, Antibiotic Resistance, and Host Adaptation

Analysis of the genome sequence of the *E. coli* SJR49 strain identified genes related to stress and survival on the same genomic island (coordinates 4305112–4806924; Figures 5D and S7; Table S6), including the chaperone genes *Hsp70* and *DnaK*, both of which are involved in the response to heat shock and cellular protection. In addition, the gene encoding SHV-2 beta-lactamase, which confers resistance to beta-lactam antibiotics, was also detected. Genes associated with genetic mobility were predicted, including *IS6* family transposase *IS26*, *IS110* family transposase *ISKpn42*, *ISL3* family transposase *ISEc38*, *IS1* protein *InsB*, and *IS5* family transposase *IS903*. The mechanisms of plasmid conjugation and transfer were evidenced by the presence of the *TraI, TraD, TraQ, TraN, TraC, TraV, TraJ, TraY, TraM*, and pilin genes. Furthermore, genes involved in plasmid segregation and stability, such as *ParM* and *StbB*, which encode plasmid segregation proteins, were identified. Finally, the presence of a DNA adenine methyltransferase and a putative DNA-binding transcriptional regulator, the DEOR-type, indicated a role in the regulation of gene expression and host adaptation.

**Figure 5:**
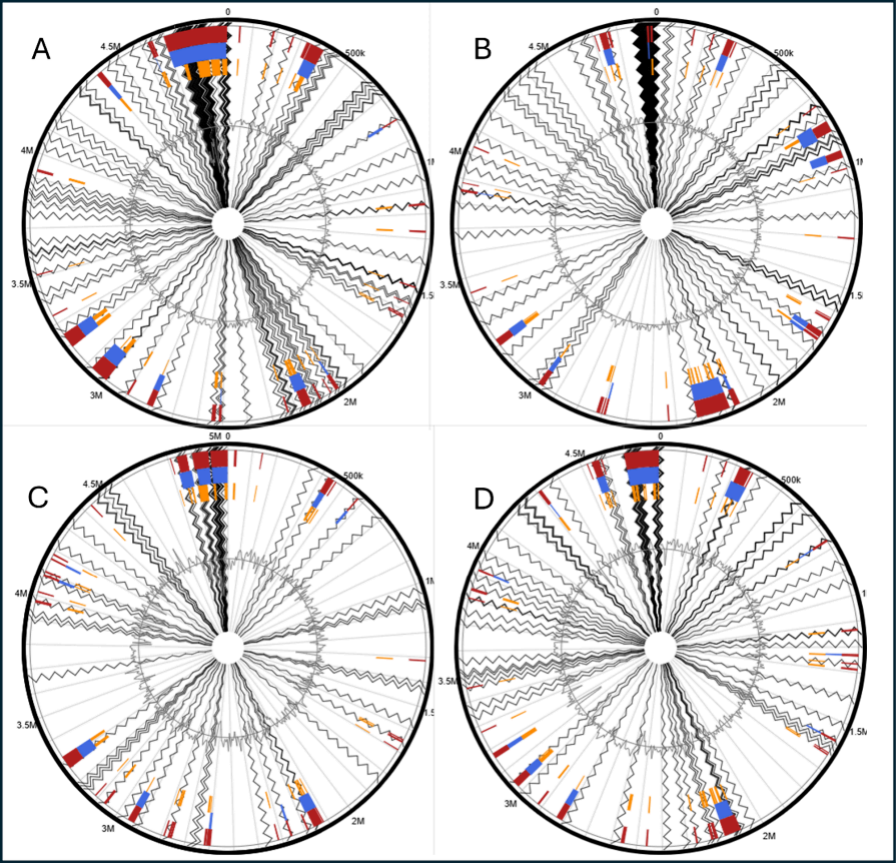
Representation of genomic islands identified in the genomes of *E. coli* isolates. Genomic circular maps for (A) SJR07, (B) SJR30, (C) SJR31, and (D) SJR49. The highlighted regions in blue represent potential genomic islands predicted by the IslandPath-DIMOB predictor tool, in yellow, the SIGI-HM tool and in red, the above-mentioned integrated tools.

The genomic sequencing analysis of *E. coli* SJR31 also revealed, in a single genomic island (coordinates 4808209–5045245; Figures 5B and S6; Table S5), genes associated with antibiotic resistance tetracycline resistance protein, class B and tetracycline repressor protein class B (of the *Tn10* transposon), beta-lactamase *TEM*, and aminoglycoside 3′-phosphotransferase and dihydropteroate synthase, which confer resistance to tetracycline, β-lactams, aminoglycosides, and sulfonamidase. Genes related to virulence included those encoding aerobactin synthase, ferric aerobactin receptor, and aerobictin (*iuc),* which are involved in iron acquisition, tyrosine recombinase (*XerC*), which is associated with the maintenance of mobile genetic elements, and toxin-antitoxin biofilm protein, ChpB toxin, antitoxin CcdA, and toxin CcdB, which are linked to biofilm formation and bacterial persistence. Furthermore, the presence of multiple transposase genes (*ISL3, IS4, IS1182, IS629, IS3, IS1, InsA, InsAB*’) as well as genes encoding replication initiation protein and transposon Tn3 resolvase were observed. In addition, genes related to environmental adaptation were identified; these included the gene encoding the c-di-GMP-binding protein, which may be involved in biofilm dispersal and F-efflux transporters. The *KpsD* and *KpsM* genes involved in capsule synthesis were also found on another genomic island.

Genes with the greatest clinical relevance when analyzing the genetic sequence of the *E. coli* SJR30 strain were present on the same genomic island (coordinates 4705238–4943081; Figures 5B and S5; Table S4). The c-di-GMP binding protein is involved in biofilm dispersal, which regulates the formation and dispersal of biofilms; toxin-antitoxin biofilm protein is associated with the maintenance of biofilms and possible cell persistence; ferric dicitrate ABC transporter (ATP binding subunit, membrane subunit, periplasmic binding protein) and ferric citrate outer membrane porin *FecA*. Major type 1 subunit fibrin (pilin), fimbria protein, and periplasmic chaperone are required for type 1 fimbriae, critical structures for adhesion and colonization of host tissues. In addition, the presence of transposases (*IS1, IS3, IS110, ISCfr6*) indicates potential genetic mobility.

In the analysis of the *E. coli* SJR07 strain (coordinates 4626467–4953318; Figure 5A), genes associated with resistance to beta-lactam antibiotics were identified, including TEM, a gene encoding a beta-lactamase, and OXA-1, a gene mediating resistance to oxacillin. In addition, genes related to resistance to aminoglycosides were detected, such as genes encoding aminoglycoside 3′-phosphotransferase and aminoglycoside N(6′)-acetyltransferase type 1. Resistance to antifolates was evidenced by the presence of genes encoding dihydrofolate reductase and dihydropteroate synthase, which are responsible for resistance to trimethoprim and sulfonamides, respectively. The gene encoding tetracycline resistance protein class C was also identified, indicating tetracycline resistance. Several genes associated with virulence have been identified, including those related to adhesion and biofilm formation, such as regulators of *fimA*, major type 1 subunit fimbrin (pilin), and fimbrial proteins. Thus, the hemin receptor gene may be involved in iron uptake. In addition, genes encoding type IV secretion system proteins, such as *VirB4, VirB9, VirB11*, and *PtlE*, which are associated with the secretion of virulence factors, were detected. Regulation of virulence gene expression may be associated with the presence of an H-NS-like DNA-binding protein gene.

This analysis also revealed the presence of mobile genetic elements and recombination factors, such as multiple transposases, including *IS1, IS2, IS3, IS6, IS21, IS66, IS110, ISL3, Tn3, TnAs1, ISEc38, ISEc76, ISKpn42,* and *IS100kyp*. The tyrosine recombinase XerC has been identified, suggesting its possible involvement in genome maintenance and recombination. In addition, the transcriptional repressor *LexA*, which is associated with stress response and regulation of mutagenesis, was detected.

None of the four strains showed the *papG* gene, which are frequently associated with bacterial invasion and adhesion in the urinary tract, within the genomic islands.

### Animal Model of Renal Infection

Table 2 shows the density of viable cells 24 h after bacterial inoculation in a murine model of renal infection (*n* = 6 per group). For strain SJR49, the log_10_ colony-forming units per gram (CFU/g) value was 7.04 at 24 h, indicating the presence of viable cells during the analysis period. Similarly, strain SJR31 showed a log_10_ CFU/g value of 6.45 at 24 h, indicating the presence of viable cells over the experimental period. In the control group, as expected, no viable cell count was observed during the analysis period (0.00 log_10_ CFU/g in 24 h), confirming the absence of microbial growth in this group.

**Table 2:** Bacterial Load in Animals Infected with the SJR49 and SJR31 Strains after 24 h (*n* = 6 per Group)

groups	Log10 CFU/g
SJR49	7.10
SJR31	6.45
Control^a^	0.00

a Uninfected mice group.

### ^99m^Tc-DMSA Renal Uptake in Mice Infected with *E. coli* SJR49 and SJR31

Ex vivo radiation counting was performed using Wizard equipment to evaluate the uptake of the radiopharmaceuticals in the kidneys of mice infected with the bacterial strains SJR49 and SJR31 (*n* = 6 per group). These results indicated a target/nontarget ratio lower than 1.0 to both groups (Figure 6), demonstrating reduced uptake of ^99m^Tc-DMSA in the infected kidney. Furthermore, the target/nontarget ratio was significantly lower in mice infected with SJR49 than in the control group (mean value = 0.6150; 95% confidence interval (CI) = 0.1084 to 1.122; *p* = 0.0245). However, the difference between the control group and mice infected with the SJR31 strain did not show statistical significance (mean value = 0.4670; 95% CI = 0.0396 to 0.9735; *p* = 0.0659). Additionally, no significant differences were observed between the groups infected with SJR49 and SJR31 (*p* = 0.5741).

**Figure 6:**
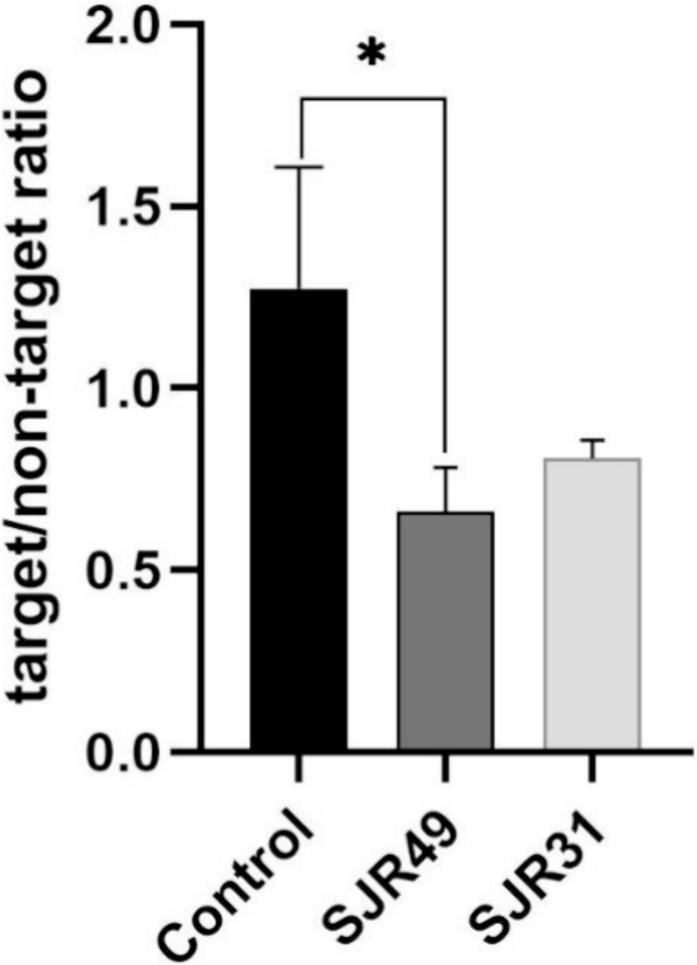
Ex vivo radiation counting after ^99m^Tc-DMSA renal uptake. The results indicate the target/nontarget ratio in the kidneys of mice infected with the bacterial strains SJR49 and SJR31 and the noninfected control group (*n* = 6 per group). Statistically significant differences (*P < 0.05). Values are expressed as mean ± standard error of the mean.

These findings are consistent with the renal scintigraphic images obtained using ^99m^Tc-DMSA. In Figure 7B, a more homogeneous radiopharmaceutical uptake between the right and left kidneys was observed, whereas in Figure 7C, the right kidney showed lower uptake than the left kidney, indicating possible functional or structural alterations resulting from the infection. As expected, in the control group (Figure 7A), no difference in radiopharmaceutical uptake was observed between the right and left kidneys.

**Figure 7:**
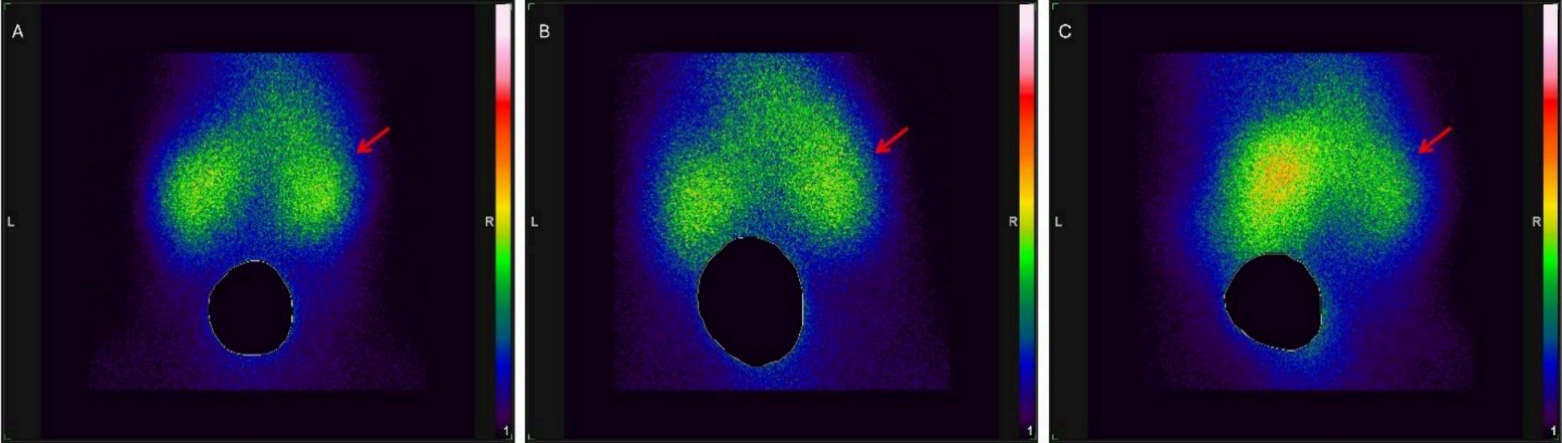
Renal scintigraphic images of mice after 20 min of ^99m^Tc-DMSA administration. (*n* = 6 per group; representative images shown). (A) Right kidney of a noninfected mouse administered saline solution. Right kidney infected with the SJR31 strain (B) and SJR49 strain (C). Red arrow indicating the right kidney.

### Histological Analysis of the Renal Tissue of Mice Infected with *E. coli* SJR31 and SJR49

Histopathological analysis of the kidneys of mice revealed marked differences between the control group and the groups infected with the bacterial strains SJR31 and SJR49 (Figure 8). In the control group, renal architecture was compromised only in the inoculation area, and discrete inflammatory infiltrates were observed only around the lesion. In contrast, the groups infected with strains SJR31 and SJR49 showed notable morphological alterations. Both groups exhibited intense and diffuse mixed inflammatory infiltrates distributed widely throughout the renal parenchyma, predominantly around the tubules. These areas were associated with extensive regions of necrosis, characterized by marked eosinophilia, loss of tissue architecture, and nuclear fragmentation. The presence of bacterial clusters was identified in the regions showing the greatest inflammatory damage, suggesting the persistence of the pathogen at the inoculation sites and its direct contribution to the inflammatory process and tissue injury. Histological data indicated that infection with strains SJR31 and SJR49 triggered an intense renal inflammatory response with severe tissue damage, whereas the control group maintained the structural integrity of the kidney with minimal changes and necrosis only at the inoculation site.

**Figure 8:**
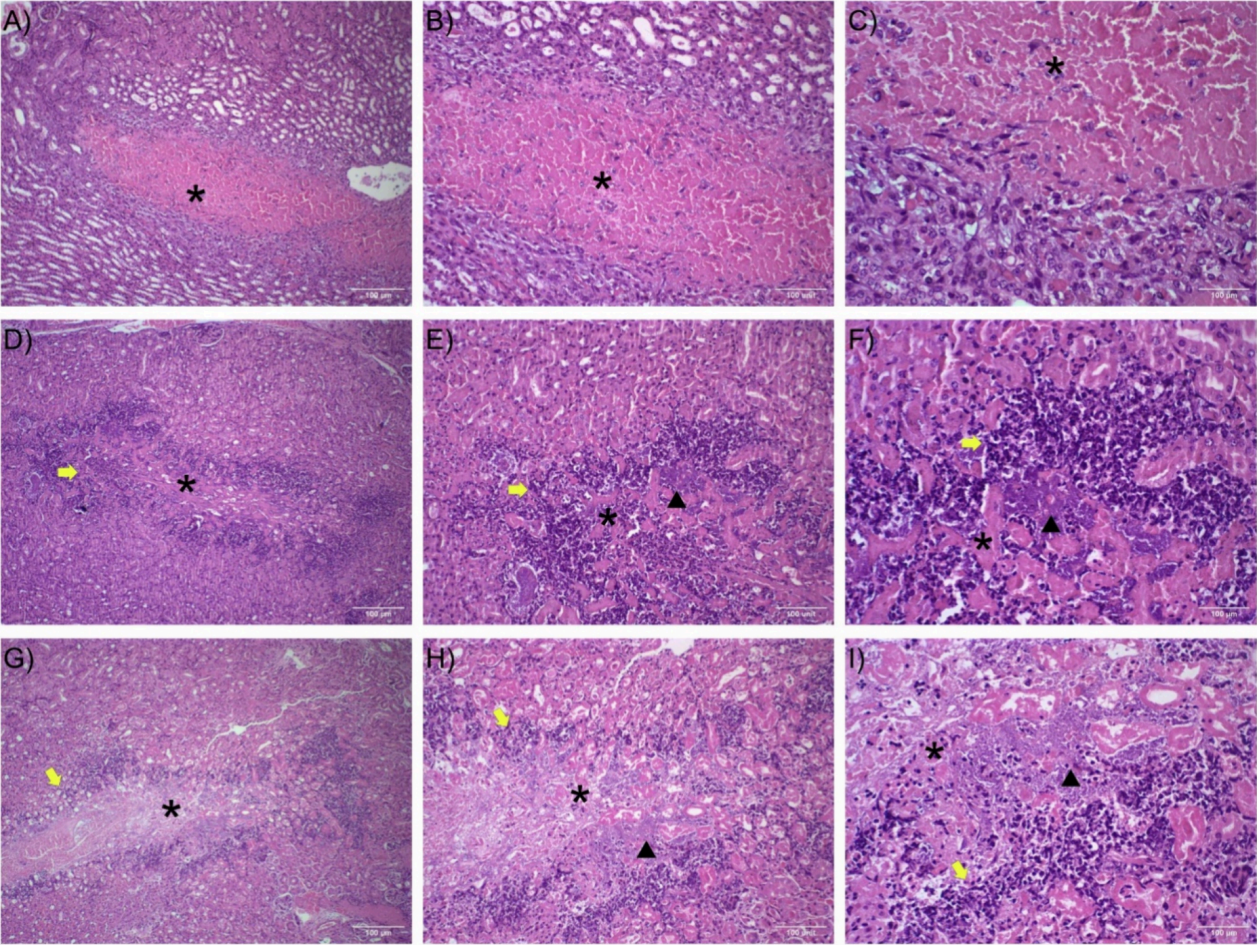
Histopathological analysis of kidneys from control mice and mice infected with *E. coli* SJR31 and SJR49 strains. Photomicrographs of mouse kidneys showing sections stained with hematoxylin and eosin. (A–C) Control group showing renal parenchyma with alterations only in the central area of the fragment at the inoculation site, with minimal inflammatory infiltration. Eosinophilic central area (necrosis) indicated by the black asterisk, refers to the site of saline inoculation, with a discrete inflammatory infiltrate and initial granulation tissue. (D–F) Group infected with strain SJR31 showing evident intense and diffuse mixed inflammatory infiltrate (yellow arrow), inflammatory cells involving tubules and the interstitium, in addition to the presence of extensive areas of necrosis (marked eosinophilia, loss of tissue architecture, black asterisk) and presence of bacterial clusters (black arrowhead). (G–I) Group infected with strain SJR49 showing alterations similar to those observed after infection with strain SJR31, including abundant and multifocal inflammatory infiltrates (yellow arrow), extensive necrosis indicated by the black asterisk, and loss of renal architecture in some areas. Bacterial clusters were also visible in regions of tissue damage (black arrowhead) (scale bar = 100 μm).

## Discussion

MLST analysis revealed genetic diversity among the clinical strains sequenced in this study, showing different STs and CCs. Strains SJR07 and SJR49 belonged to the ST10 CC, indicating a close evolutionary relationship and possible clonal dissemination in the studied environment.

CC10 is recognized as one of the most widespread *E. coli* CCs, and is commonly identified in both community and hospital settings. A large-scale study conducted by Manges et al.,^7^ which analyzed hundreds of extraintestinal *E. coli* (ExPEC) isolates from different geographic regions, showed that ST10 was among the most frequently detected, regardless of location. Strains related to ST10 have been shown to be involved in UTIs and invasive infections such as bloodstream infections; they have also been identified in commensal contexts, and are frequently associated with antimicrobial resistance. CC10 has also been isolated from a wide variety of sources, including food, animals, and environmental samples, highlighting its ecological versatility and potential for zoonotic and environmental transmission.^8^ The detection of CC10 in this study reinforces its epidemiological relevance, and may reflect its capacity for dissemination in different contexts.

Small variations in alleles, such as those observed in *purA* (allele 8 in SJR07 and allele 13 in SJR49), indicate that these strains may have undergone mutations or recombination events over time.^9^ Strain SJR30 was classified in the ST448 CC and SJR31 in the ST354 CC, showing greater genetic divergence than the other strains. According to other studies, variations in alleles across the seven loci of the MLST scheme may explain the presence of multiple strains circulating in the studied population, which possibly adapted to different selective pressures or environments.^9^ The study by Manges et al. also identified ST354 as one of the 20 major *E. coli* ExPEC lineages globally, which is frequently associated with extraintestinal infections and resistance to multiple antimicrobial drugs. This ST has already been detected in various clinical and environmental contexts, including clusters of ESBL-producing isolates from humans and water sources. Therefore, the identification of strain SJR31 as belonging to ST354 may reflect its environmental adaptability and epidemiological relevance. Moreover, phylogenomic comparison with contemporary Brazilian and international UPEC isolates revealed that SJR31 clusters closely with recent ST354 strain PDT010099056 from Brazil, indicating the long-term persistence and dissemination of this lineage in the region. This finding suggests that some genomic features, including virulence and resistance determinants, have been maintained over time, supporting the hypothesis of local adaptation and successful circulation of ST354 among both community and healthcare-associated infections.

The phylogenetic analysis performed in this study complements these findings by revealing that the isolated strains belonged to different phylogenetic groups, reinforcing the genetic variability within the studied population. Strains SJR30 and SJR31 were classified into phylogenetic groups B1 and F, respectively. However, strains SJR49 and SJR07 were grouped in phylogenetic group A, forming the same clade, which indicates a recent common ancestor and a closer evolutionary relationship. Furthermore, phylogroup B1 was frequently associated with commensal and community-acquired strains, although strain SJR30 did not show close relatedness to previously reported global strains. Strain SJR31 clustered near the global ST354 strain PDT001090596, which has been reported as an ExPEC linked to both community and hospital infections. Strains SJR49 and SJR07 were grouped together with the international lineages from CC 10, L1LB, which was isolated in Peru, and thestrain PDT001089438, both of which are widely distributed and recognized for their versatility in colonizing humans, animals, and the environment. The presence of these phylogroups, including lineages often found beyond hospital settings, underscores the genetic relatedness of our isolates to strains with a broad epidemiological distribution, spanning from community reservoirs to healthcare-associated niches. These results provide information for better characterization of pathogenic *E. coli* recovered from different isolation sources and may facilitate the development of better tools to identify potential contamination transmission routes.^10^

Genomic analysis of the clinical isolates of UPEC SJR07, SJR30, SJR31, and SJR49 revealed a heterogeneous profile of genes related to antimicrobial resistance, suggesting that the strains present distinct resistance determinants in comparison with other isolates previously described in the literature. Among the identified genes, those related to resistance to aminoglycosides stood out, such as *aac­(3)-IId, aac­(6′)-Ib-cr, and aph(3″)-Ib*. The *aac­(3)-IId* gene, which was found in strain SJR31, encodes an acetyltransferase that modifies aminoglycosides and confers resistance to antibiotics such as gentamicin and tobramycin.^11^ The *aac­(6′)-Ib-cr* variant, which was identified in strain SJR07, is clinically relevant because it confers resistance to both aminoglycosides and fluoroquinolones.^11^ The *aph­(3*″*)-Ib* and *aph­(6)-Id* genes, detected in strains SJR31 and SJR07, encode phosphotransferases that inactivate aminoglycosides through phosphorylation.^12^ Among the genes related to β-lactam resistance, important β-lactamases were identified in SJR49 chromossome (SHV-2) and plasmid (SHV-12). Thesevariants,, are capable of hydrolyzing the β-lactam ring and is frequently associated with self-transmissible plasmids, which carry genes for resistance to other drug classes and are widely distributed globally among Enterobacteriaceae, emphasizing its clinical importance.^13^ SHV-2 is an extended-spectrum beta-lactamase (ESBL) found frequently in *E. coli*, Klebsiella pneumoniae, and Shigella flexneri. SHV-12 is an ESBL found frequently in *Acinetobacter baumannii*. The co-occurrence of these two SHV variants in the same strain highlights the potential for broad-spectrum β-lactam resistance and underscores the clinical relevance of monitoring plasmid-borne ESBLs in pathogenic Enterobacteriaceae.

The EC-15 enzyme, which was also found in strain SJR49, has similar β-lactamase activity, conferring resistance to various β-lactams.^14^ The TEM-1 gene, which was identified in strains SJR31 and SJR07, encodes one of the most prevalent β-lactamases, conferring resistance to penicillins and some cephalosporins.^15^ The EC-13 gene, which encodes another β-lactamase from the EC family, was found in strain SJR030.

Resistance to sulfonamides was observed with the presence of the *sul2* gene in strains SJR31 and SJR07; this gene encodes a variant of dihydropteroate synthase that is insensitive to inhibition by sulfonamides.^16^ The *tet­(B)* gene, responsible for tetracycline resistance through an active efflux mechanism, was identified in strain SJR03.^17^ In addition, the *dfrA17* gene, found in strain SJR31, encodes a dihydrofolate reductase resistant to trimethoprim, which is often associated with class 1 integrons.^18^ The SJR07 strain also harbored the *mdtK* gene, which encodes a multidrug efflux pump and contributes to resistance to several antimicrobials, including fluoroquinolones.^17^ None of the clinically relevant resistance genes identified in the UPEC strains in the present study were found in *E. coli* K12, which was used as a control. In contrast, CFT073 harbored the resistance genes *EC-5* and *TEM-181.*

Another relevant finding of this study was the identification of resistance genes located on plasmids in all isolates. The plasmids from strains SJR07 and SJR31 presented resistance genes, including *dfrA17*, *tet­(B)*, *sul2*, APH­(3′)-IB, and APH(6’)-ID in SJR07, and OXA-1, *tet­(A),* and AAC(6′)-IB-CR in SJR31. The TEM-1 gene, which is widely known for its association with β-lactam resistance, was detected in both strains. The presence of plasmids in all the analyzed strains is clinically relevant, since it represents a genetic reservoir with the potential to acquire and transfer new resistance determinants through conjugation. This potential for horizontal dissemination among bacteria, including those of different species, contributes to increased resistance in hospital environments and increases the risk of therapeutic failure. Additionally, some plasmids carry virulence genes or hitherto undescribed genetic elements, reinforcing the need for continuous surveillance, even for seemingly “silent” plasmids.^19,20^ Therefore, these findings reinforce the importance of genomic monitoring of antimicrobial resistance in UPEC strains, highlighting not only the existing genetic variability but also the role of plasmids as important vehicles in the dissemination of resistance genes in clinical settings.

Genomic analysis of clinical *E. coli* UPEC strains revealed the presence of genes related to antimicrobial resistance as well as virulence, even without a common genetic signature that characterizes classical uropathogenic pathogens. In the analysis based on the VFDB, virulence determinants presented a heterogeneous profile among strains SJR07, SJR30, SJR31, and SJR49, as well as in comparison with isolates described in the literature. Among the most prominent virulence genes were pseudopilins (*gps*), which are responsible for the secretion of proteins and virulence factors through the outer membrane of Gram-negative bacteria and are present in all studied strains and in the control strain CFT073. Other notable genes included those related to iron uptake, such as aerobactin (*iuc*) and capsule genes (*Kps*), found in SJR31 and CFT073. Strain SJR49 stood out for containing antiphagocytosis genes (*KP1*) and secretion system genes (*VIPB* and *Tss*), indicating a virulent arsenal with the potential for immune evasion.^21–23^

Investigation of genomic islands revealed functional clustering of genes associated with virulence, antimicrobial resistance, genetic mobility, and environmental adaptation, reinforcing the role of these regions in the evolution and genomic plasticity of UPEC. Strain SJR49 presented, on a single genomic island, genes such as *Hsp70* and *DnaK* (encoding chaperones involved in the thermal stress response), the beta-lactamase gene *SHV-2*, and multiple transposases (*IS6, IS110, ISL3, IS1, IS5*), as well as genes involved in the plasmid conjugation system (*Tra* and *Pilin*), segregation (*ParM, StbB*), and gene regulation (DNA adenine methyltransferase and DEOR-type regulator).

In strain SJR31, the genomic island contained multiple resistance genes, including those encoding TEM and tetracycline resistance proteins, APH(3′), and dihydropteroate synthase, which were associated with resistance to aminoglycosides and sulfonamides associated with transposon *Tn10*. Virulence genes included those encoding aerobactin and its receptor as well as elements linked to biofilm formation and persistence, such as *ChpB, CcdA*, and *CcdB*. The presence of various transposable elements (*ISL3, IS4, IS1182*, etc.), replication proteins, and adaptation genes, such as those encoding the c-di-GMP-binding protein and efflux transporters, indicates high plasticity and adaptive potential.

The analysis of strain SJR30 revealed a genomic island with genes associated with biofilm formation and maintenance (c-di-GMP binding protein and toxin-antitoxin proteins), iron transport (*fec operon*), and adhesion (fimbriae, periplasmic chaperones), as well as transposases indicating genetic mobility. These findings indicate pathogenic potential related to persistent colonization of the urinary tract.

Strain SJR07 harbored genes associated with resistance to beta-lactams (TEM, OXA-1), aminoglycosides (APH(3′), AAC(6′)), antifolates (dihydrofolate reductase and dihydropteroate synthase), and tetracyclines. Virulence genes related to adhesion and biofilm formation, such as *fimA* and the genes encoding fimbriae and hemin receptors, were also detected. Notably, genes from the type IV secretion system (*VirB4, VirB9, VirB11, PtlE*) were found along with regulatory elements, such as the DNA-binding protein H-NS. The presence of a wide variety of transposases and the recombinase *XerC* reinforced the role of these genomic islands in bacterial recombination and evolution.

A study of 557 *E. coli* isolates demonstrated that the genetic diversity observed in these strains could be attributed to homologous recombination events and point mutations. These processes contribute substantially to the genetic variation within bacterial populations, even in restricted geographic contexts. Analysis of the strains revealed variations in the frequency and recombination hotspots, indicating that different lineages may have distinct propensities to exchange genetic material.^24^ These data reinforce the concept that UPEC strains possess a highly dynamic genome, with genomic islands harboring resistance, virulence, mobility, and environmental adaptation genes. This plasticity contributes to bacterial persistence in hostile environments, such as the human urinary tract, and under antimicrobial selective pressure, posing a major challenge to the clinical management of UTIs.^25^

The results from the renal infection murine model demonstrated that both *E. coli* strains could colonize the renal tissue and remain viable throughout the experimental period. The recovery of viable bacterial cells from the kidneys indicated that these strains possess mechanisms that promote their survival in the renal environment, even in the presence of the host’s initial defense responses.

The persistence of the strain SJR49 in the murine model was consistent with the genomic findings. as it presented antiphagocytosis genes and type VI secretion genes, which were also concentrated upon a functional genomic island. These factors may have favored colonization and immune evasion, explaining the persistence in the urinary tract.^26^

Complementing these findings, the results for ^99m^Tc-DMSA renal uptake reinforced the microbiological data by showing functional alterations in infected kidneys. A target/nontarget ratio equal to 1 indicates no difference in ^99m^Tc-DMSA uptake between the right and left kidneys, reflecting normal renal function. On the other hand, a ratio lower than 1 reflects reduced uptake of the radiopharmaceutical in the infected kidney relative to the contralateral kidney, indicating functional impairment. The reduced target/nontarget ratio in the infected groups, especially in animals infected with strain SJR49, indicated functional impairment of the affected kidney. The statistically significant reduction in ^99m^Tc-DMSA uptake in the SJR49 group in comparison with that in the control group indicates a greater degree of renal damage or dysfunction. However, the reduction observed in the SJR31 group was not significant.

Scintigraphic images corroborated these quantitative data, demonstrating asymmetry in the uptake between the right kidneys and left kidneys of the same animal infected with strain SJR49; the same findings were also observed in comparison with the right kidney of the control animals. This asymmetry supports the hypothesis that SJR49 has a greater pathogenic capacity or tissue damage-inducing potential. This difference between strains may reflect variations in their virulence profiles, including factors associated with tissue invasion or the degree of the induced inflammatory response.

Histological analysis confirmed these functional alterations, revealing an intense and diffuse renal inflammatory response accompanied by extensive areas of necrosis and the presence of bacterial clusters within the parenchyma. These changes indicate that both strains were capable of inducing a substantial inflammatory process with tissue destruction, reinforcing their pathogenic potential in the development of acute renal injury. Although genomic data did not reveal the presence of the papG operon, these genes are frequently found in strains isolated from patients with clinical pyelonephritis.^27^ This genomic profile is consistent with the results of the phylogenetic analyses, which placed the isolates at a distance from the globally disseminated ST69 lineage, a high-risk clone well-known for harboring such virulence determinants within mobile genetic elements. The absence of these genes in the genomic islands may indicate that the studied strains rely on alternative mechanisms of colonization and adaptation and are more closely related to community-associated lineages than to hospital-restricted pathogenic clones such as ST69 and ST131. This highlights a distinct epidemiological context wherein the isolates may circulate with potentially lower pathogenic potential in the urinary tract in comparison with classical ExPEC high-risk clones, but still maintain genetic relatedness to globally distributed lineages such as ST10 and ST354.

The distinct histological findings for the infected and control groups reinforced the validity of the experimental model and highlighted the pathological effects of renal colonization by these strains. In particular, tissues infected with SJR49 showed greater necrosis area and granulation tissue, which was consistent with the lower ^99m^Tc-DMSA uptake, indicating a more aggressive infectious profile.

Taken together, these results demonstrate that strains SJR49 and SJR31 can persist in renal tissue and induce substantial functional and histopathological changes. Although both strains caused renal damage, strain SJR49 appeared to induce a more severe response in the host. These findings are relevant to our understanding of bacterial persistence in UTIs and highlight the clinical importance of virulence-associated genetic traits in determining infection outcomes. While these insights advance our understanding of UPEC pathogenic mechanisms, this study is limited by the small number of isolates analyzed from a single region and time frame, and its conclusions should be viewed as exploratory. Even so, the identified resistance and virulence determinants offer valuable clues for therapeutic innovation, potentially guiding drug discovery efforts targeting mechanisms of renal colonization and persistence. Expanding these analyses through large-scale genomic surveillance could further support the development of precision antimicrobial and antivirulence strategies.

## Conclusions

This study provides evidence of the genetic diversity and adaptive potential of the UPEC isolates analyzed, which displayed a heterogeneous repertoire of antimicrobial resistance and virulence genes located on plasmids and genomic islands. The persistence and pathogenicity observed in the experimental kidney infection model, particularly for strain SJR49, suggest a possible link between these genetic determinants and renal colonization and tissue damage. Although limited to four isolates from a single geographic and temporal source, the results offer valuable insights into the molecular basis of UPEC virulence and resistance, generating hypotheses that may guide future studies and support the design of strategies to control multidrug-resistant and highly virulent UPEC in clinical settings.

## Material and Methods

### Bacterial Isolates

Four clinical isolates of quinolone-resistant UPEC (SJR49, SJR31, SJR30, and SJR07) were kindly provided by the Clinical Microbiology Laboratory of the Universidade Federal de São João Del Rei, Divinópolis, MG, Brazil. All isolates were recovered from the urine of women with clinical and laboratory diagnoses of community- or hospital-acquired CA-UTI between May and November 2009 (State of Minas Gerais, Brazil). The isolates were originally described by Cristina de Paiva and colleagues (2012) and were collected under ethical approval from the Ethics Committee of the Federal University of Minas Gerais (protocol 178/09). Bacterial identification was previously performed using the Vitek2 automated system (bioMérieux, Hazelwood, MO), biochemical assays (Rugai Phoenix), and morphological methods (Gram and MacConkey agar). The susceptibility profile to various antimicrobials was determined in vitro by the Kirby-Bauer assay, as shown in a previous study.^28^

All microorganisms were stored in ultrafreezer stocks at −80 °C in nutrient broth (Kasvi, Spain) containing 20% glycerol (cryoprotectant). For analytical procedures, the cultures were reactivated in Mueller–Hinton (MH) broth and after 24 h of incubation at 37 °C, streaks of each isolate were performed on Mueller–Hinton agar (Kasvi, Spain) and kept in a refrigerator at 4 °C. The refrigerator stock was maintained every 15 days, with a maximum of three passages allowed; thus, the refrigerator stock was used within 45 days after reactivation.^29^ Selective pressures on the strains were applied by adding the antibiotic ciprofloxacin at concentrations based on CLSI 2018 breakpoint values to both the stock broth and the agar used for making the streaks.

### Animal Model of Renal Infection

The in vivo experiments were performed in Balb/c mice weighing approximately 25 g and approximately 8 weeks of age, obtained from the central animal facility of UFMG, Belo Horizonte, MG, Brazil. The animals were housed together (6 animals per box) in polyester boxes at the animal facility of the radioisotope laboratory of the Faculdade de Farmácia, UFMG. During the study, the mice had a 12-h light-dark cycle at a temperature of 23 °C, and received food and water ad libitum. The UFMG Animal Ethics Committee approved this study (Protocol CEUA 215/2024).

The mice were randomly divided into three groups: group 1 was infected with *E. coli* SJR49; group 2 was infected with *E. coli* SJR31; and group 3 contained uninfected controls. All animals in the three groups underwent the surgical procedure. Randomization was performed by an independent researcher. The animals were placed in cages and randomly numbered, without prior knowledge of which cage corresponded to each experimental group. Mice were acclimated in the animal facility for 15 days before the surgical procedure. All surgeries were performed by the same researcher under identical conditions for all animals. The investigators responsible for the scintigraphic and histopathological analyses were blinded to the experimental group allocation. Animals were excluded from the quantitative analysis of renal function in the following cases: (i) loss of the animal during the anesthesia process for image acquisition, preventing the administration or proper distribution of the radiopharmaceutical; (ii) failure in the administration of the radiopharmaceutical into the tail vein, resulting in discrepant data.

For the surgical procedure, which was adapted from the study by Grupta et al. (2017), 100 μL of the bacterial inoculum were administered at a concentration of 10^8^ CFU/mL in the right kidney. The control group received 100 μL of 0.9% w/v saline. At 24 h after the surgical procedure, the animals underwent kidney scintigraphy and were euthanized for kidney and blood collection.^30^

### Scintigraphic Imaging

Radiolabeling of dimercaptosuccinic acid (DMSA) with the technetium-99m (^99m^Tc) radioisotope was performed as described in the kit package insert (reference to the package insert). Briefly, a lyophilized kit containing 1 mg of DMSA and 0.44 mg of SnCl_2_·2H_2_O at a pH of 7.0 was reconstituted with 1.0 mL of sodium pertechnetate (Na^99m^TcO_4_) solution, showing 74 MBq of activity, obtained from a ^99^ Mo/^99m^Tc generator (Ipen, São Paulo, SP, Brazil). After the addition of Na^99m^TcO_4_, the vial was kept in an upright position at room temperature for 30 min to complete the radiolabeling process.

For image acquisition, previously anesthetized mice received 100 μL of ^99m^Tc-DMSA (activity, 5.9 MBq) intravenously through the tail vein. Twenty minutes after administration, images were acquired using a single-head gamma camera equipped with a low-energy pinhole collimator (Nuclide TH22; Mediso, Hungary). During the procedure, the animals were positioned in the prone position, and image acquisition was performed over a period of 10 min using a 256 × 256 × 16-pixel matrix, with a ±10% energy window centered at 140 keV.^6^

#### Quantitative Analysis of Renal Function

After image acquisition, the animals were euthanized, and the kidneys were collected for radioactivity quantification using a gamma counter (Gamma Counter Wizard 1470, PerkinElmer).^31^ The percentage of radioactivity per gram of tissue (% ID/g) was determined for each organ. A dose standard was used to correct radioactive decay related to ^99m^Tc. The (% ID/g) of each organ was calculated by dividing the radioactive activity per gram of tissue by the standard dose.^32^ After quantifying the uptake of ^99m^Tc-DMSA by the infected kidneys, the target-to-nontarget ratio was determined. The target/nontarget ratio of the infected (right) and contralateral (left) kidneys was calculated as follows:

Target‐to‐non‐targetratio=(%dose/gofinfectedkidney)/(%dose/gofcontralateralkidney)

#### Bacterial Load

Twenty-four hours after inoculation of animals with bacterial suspensions, mice were euthanized by cervical dislocation under anesthesia. The kidneys were then collected, washed with sterile saline solution, homogenized, and subjected to serial dilutions (10^–1^–10^–5^) in saline solution (0.9% NaCl). The samples were plated on Muller–Hinton agar (Kasvi, São José dos Pinhais, PR, Brazil) and incubated at 37 °C for 24 h. After incubation, the CFUs were quantified for each sample.^33^

### Histopathological Analysis

After euthanasia, the right kidney of each animal was collected to assess possible cellular damage and identify bacterial foci. The tissue samples were fixed in 10% neutral buffered formalin for 3 days, gradually dehydrated in ethanol, cleared in xylene, and embedded in paraffin blocks. The blocks were sectioned at a thickness of 4 μm and mounted on microscope slides. The slides were stained with hematoxylin and eosin (H&E) and analyzed by two experienced pathologists who were blinded to the study.

### Statistical Analysis

The Kruskal–Wallis test followed by Dunn’s multiple comparisons test was used to compare tissue radioactivity between infected groups with different bacterial strains and the uninfected control. All statistical analyses were performed using GraphPad Prism 5.03 (GraphPad Software Inc., La Jolla, CA), and p values <0.05 were considered statistically significant.

### DNA Extraction and Genomic DNA Sequencing

Total DNA was extracted from the four *E. coli* UPECs isolates using the Wizard Genomic DNA Purification Kit (Promega) in accordance with the manufacturer’s instructions. Whole-genome shotgun libraries were prepared using an Illumina TruSeq DNA Sample Preparation Kit. Genomic DNA libraries were sequenced using a HiSeq 2500 platform (Illumina) with paired-end reads (2 × 150 bp) and a 450-bp insert size.

### Quality Analysis and Assembly

The base quality of fastq files was checked, and low-quality reads (Phred <30) were filtered using Fastp v0.24.0.^34^ The assembly was performed de novo using SPAdes v4.0.0 and Skesa v2.3.0, and the completeness and contamination of the assemblies were estimated using CheckM2 v1.0.2 tools (Table S1).^34,35^ The four assemblies were deposited in the NCBI Genome database under Bioproject PRJNA1311089 and accession numbers JBQWFO000000000 (SJR07), JBQWFP000000000 (SJR30), JBQWFQ000000000 SJR31 and JBQWFR000000000 (SJR49)

### Public Genomes Used in This Study

Public data sets of 82 complete genomes of *E. coli* strains were downloaded from the NCBI RefSeq genome database (accessed on October 2024) for comparative analysis, including phylogenetic, virulence, and resistance gene prediction. The accession numbers for all public genomes are shown in Table S2.

### Strain Typing and Taxonomic and Phylogenetic Analysis

ANI analysis was performed to confirm the taxonomy of the isolates at the species level using the JSpeciesWS genome database.^36^ Pairwise alignments of genomes with ANI values >95% in the database were considered to belong to the same species. *E. coli* subtyping was performed using MLST based on the alleles *adk, fumC, gyrB, icd, mdh, purA*, and *recA* using public databases for molecular typing and the microbial genome diversity—pbMLST tool.^37^ The concatenated sequences of the alleles described above from each genome were used to perform a phylogenetic analysis based on the maximum-likelihood method with 1000 bootstrap iterations. The phylogenetic results were further confirmed by performing a core-genome SNP based alignment among the four isolates and representative genomes from global strains and each phylogroups. The genome of *Escherichia fergusonii* FDAARGOS 1499 (NZ_CP083638.1) was used as the outgroup in both MLST and Core-genome phylogenetic analysis.The extraction and alingment of the core-genome was performed using the software ppanggolin v.1.2.105.

### Identification of Virulence and Resistance Genes

To identify antibiotic resistance and virulence factor genes, the predicted amino acid sequences of all genomes were aligned to the databases CARD (version 4.0.1, released on May 29, 2025) and VFDB (released in June 2025), using the Panvita tool v. 1.1.9. Alignments showing ≥70% sequence identity and 100% coverage were considered homologous to reference genes. The results were visualized as heatmaps generated using the pheatmap package (v1.0.12) available for RStudio v.4.5.0.

## Supplementary Material



## Data Availability

The four assemblies and SRA were deposited in the NCBI Genome database under Bioproject PRJNA1311089. All output tables files from genomic analysis are available at 10.6084/m9.figshare.30439109.

## Notes

Antimicrobial resistance genes identified in the analyzed *Escherichia coli* genomes; virulence genes identified in the analyzed *Escherichia coli* genomes; core-genome SNP phylogenetic tree based on representative genomes of different *Escherichia coli* phylogroups and globally disseminated clonal complex strains; genomic island boundaries and tRNA positions predicted in *E. coli* SJR07; genomic island boundaries and tRNA positions predicted in *E. coli* SJR30; genomic island boundaries and tRNA positions predicted in *E. coli* SJR31; genomic island boundaries and tRNA positions predicted in *E. coli* SJR49; quality and assembly metrics of the analyzed genomes; UPEC isolates plasmid typing; genomic island boundaries and tRNA coordinates in *E. coli* SJR07; genomic island boundaries and tRNA coordinates in *E. coli* SJR30; genomic island boundaries and tRNA coordinates in *E. coli* SJR31; genomic island boundaries and tRNA coordinates in *E. coli* SJR49; Newick file for MLST-based tree; and Newick format for Coregenome-based tree (PDF)

## Abbreviations

ANI: average nucleotide identity
CARD: comprehensive antibiotic resistance database
CFU: colony forming units
MLST: multilocus sequence typing
ST: sequence type
CC: clonal complex
UPEC: uropathogenic *E. coli*
UTI: urinary tract infection
VFDB: virulence factor database
