# Supplementary material for: Genomic and In Vivo Characterization of Antimicrobial Resistance and Virulence in UPEC Escherichia coli Isolated from Brazilian Cases of UTI
Source: ACS Omega. 2025 Nov 12;10(46):55554–66. doi: 10.1021/acsomega.5c06488 (PMC12658690; doi:10.1021/acsomega.5c06488)
Supplement: Supplementary file 1 [file ao5c06488_si_001.pdf]

Supporting Information:

Genomic and In Vivo Characterization of Antimicrobial Resistance and Virulence in UPEC Escherichia coli Isolated from Brazilian Cases of UTI

Barbara Gatti Cardoso[1], Rodrigo Dias de Oliveira Carvalho[1], Bertram Brenig[2], Vasco Azevedo[3], Marisa Salvi[4], Geovanni Dantas Cassali[4], Simone Odilia Antunes Fernandes[1], Valbert Nascimento Cardoso*[1]

[1]Universidade Federal de Minas Gerais, Departamento de Análises Clínicas e Toxicológicas, Faculdade de Farmácia, Laboratório de Radioisótopos, Belo Horizonte, MG, BR 31270-901

[2]I Institute of Veterinary Medicine, Department of Molecular Biology, Göttingen, DE

[3]Universidade Federal de Minas Gerais, Departamento de Genética, Ecologia e Evolução, Instituto de Ciências Biológicas, Laboratório de Biologia Celular e Molecular, Belo Horizonte, MG, BR 31270-901.

[4]Universidade Federal de Minas Gerais, Departamento de Patologia geral, Instituto de Ciências Biológicas, Belo Horizonte, MG, BR 31270-901

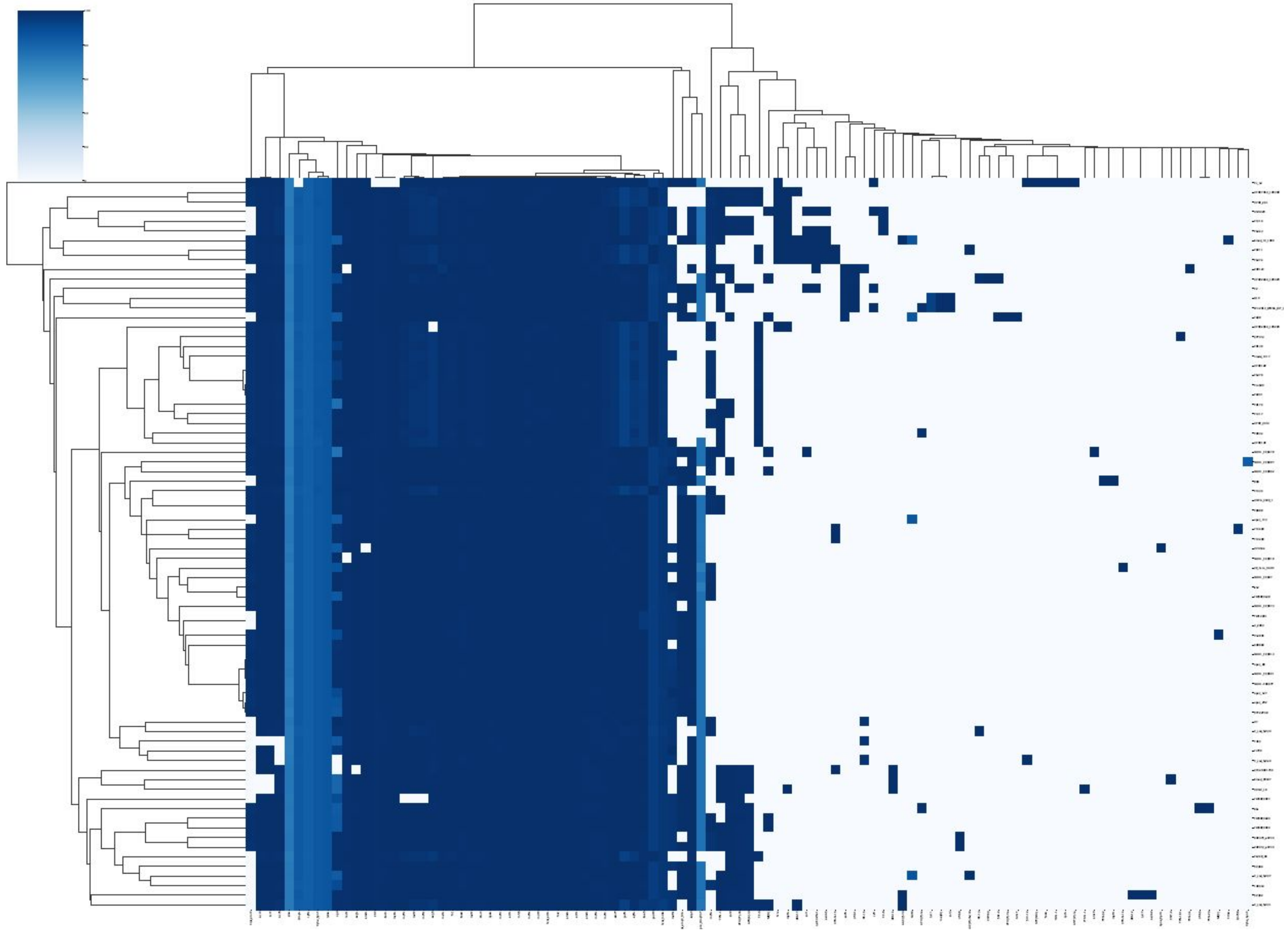

Figure S1. Antimicrobial resistance genes identified in the analyzed *Escherichia coli* genomes. Amino acid sequences were aligned against the Comprehensive Antibiotic Resistance Database (CARD). Sequences showing at least 70% identity and 100% coverage were considered

homologous. The presence or absence of resistance genes is represented in a heatmap, with each row corresponding to a gene and each column representing an *E. coli* strain.

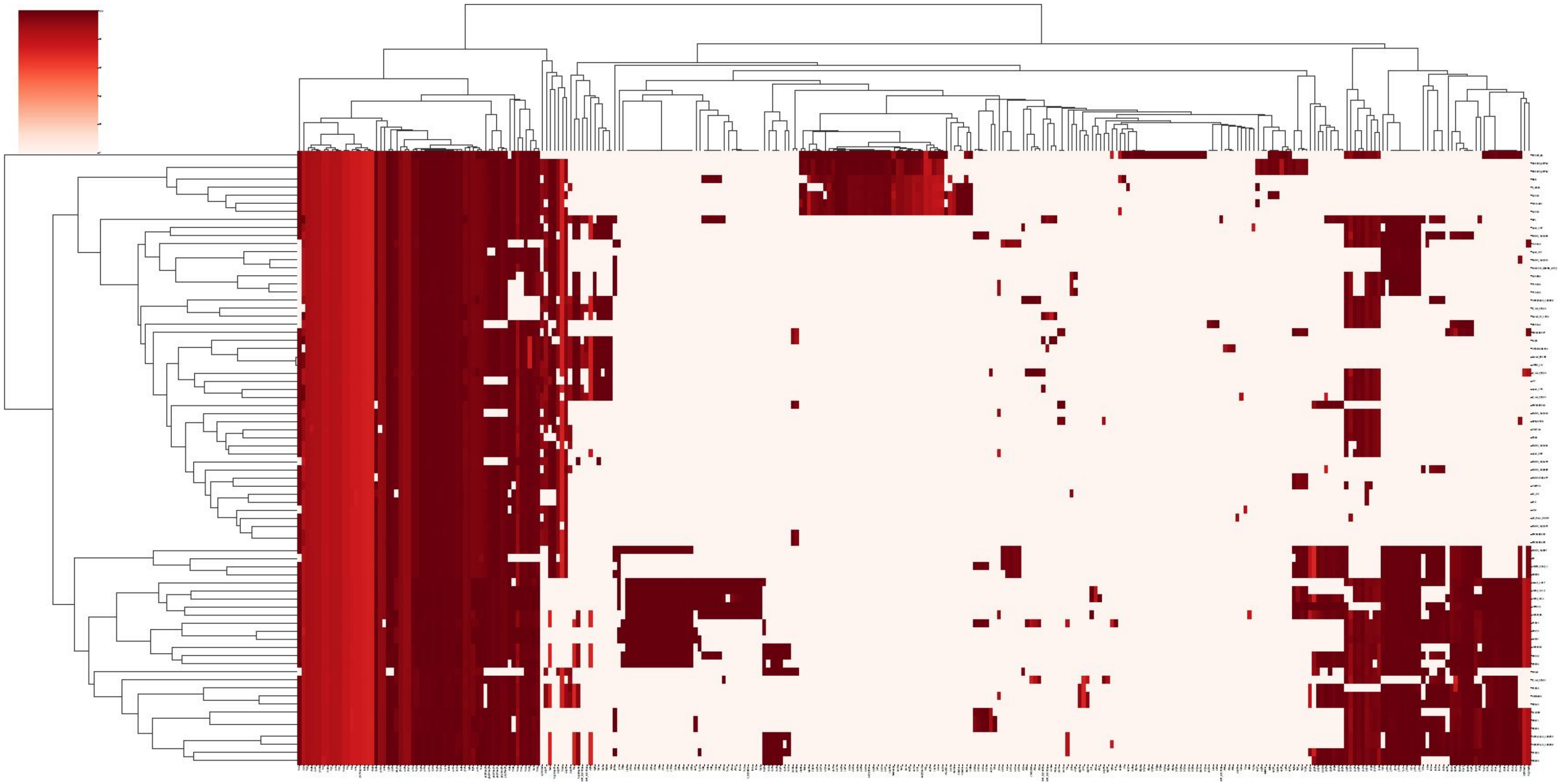

Figure S2. Virulence genes identified in the analyzed Escherichia coli genomes. Amino acid sequences were aligned against the Virulence Factor Database (VFDB), considering as homologous those with at least 70% identity and 100% coverage. The presence or absence of virulence genes is shown in a heatmap, where rows represent genes and columns correspond to the different E. coli strains analyzed

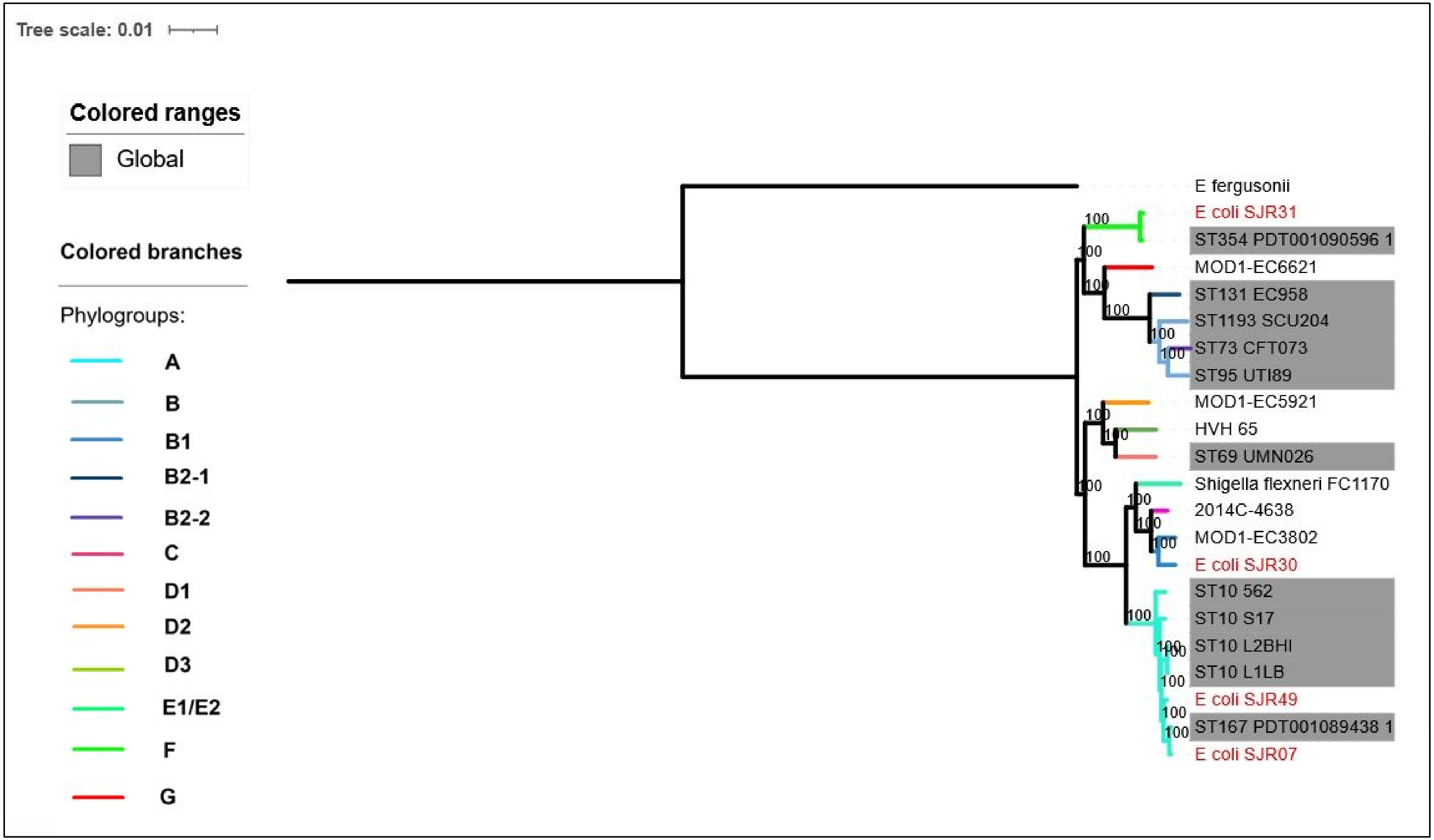

Tree scale: 0.01
Colored ranges
Global
Colored branches
Phylogroups:
A
B
B1
B2-1
B2-2
C
D1
D2
D3
E1/E2
F
G
E fergusonii
E coli SJR31
ST354 PDT001090596 1
MOD1-EC6621
ST131 EC958
ST1193 SCU204
ST73 CFT073
ST95 UTI89
MOD1-EC5921
HVH 65
ST69 UMN026
Shigella flexneri FC1170
2014C-4638
MOD1-EC3802
E coli SJR30
ST10 562
ST10 S17
ST10 L2BHI
ST10 L1LB
E coli SJR49
ST167 PDT001089438 1
E coli SJR07

Figure S3. Core-genome SNP phylogenetic tree based on representative genomes of different *Escherichia coli* phylogroups and globally disseminated clonal complex strains. Phylogenetic relationships among *E. coli* isolates based on the concatenated alignment of Core-genome (2627 genes; total length = 2.603.157 bp). The tree was inferred using the maximum-likelihood method under the GTRGAMMA evolutionary model with 1,000 bootstrap replicates. Bootstrap support values are indicated by red dots on branch bipartitions. Phylogroups are shown on the left panel, distinguished by different colors. The genome of *Escherichia fergusonii* was used as an outgroup.

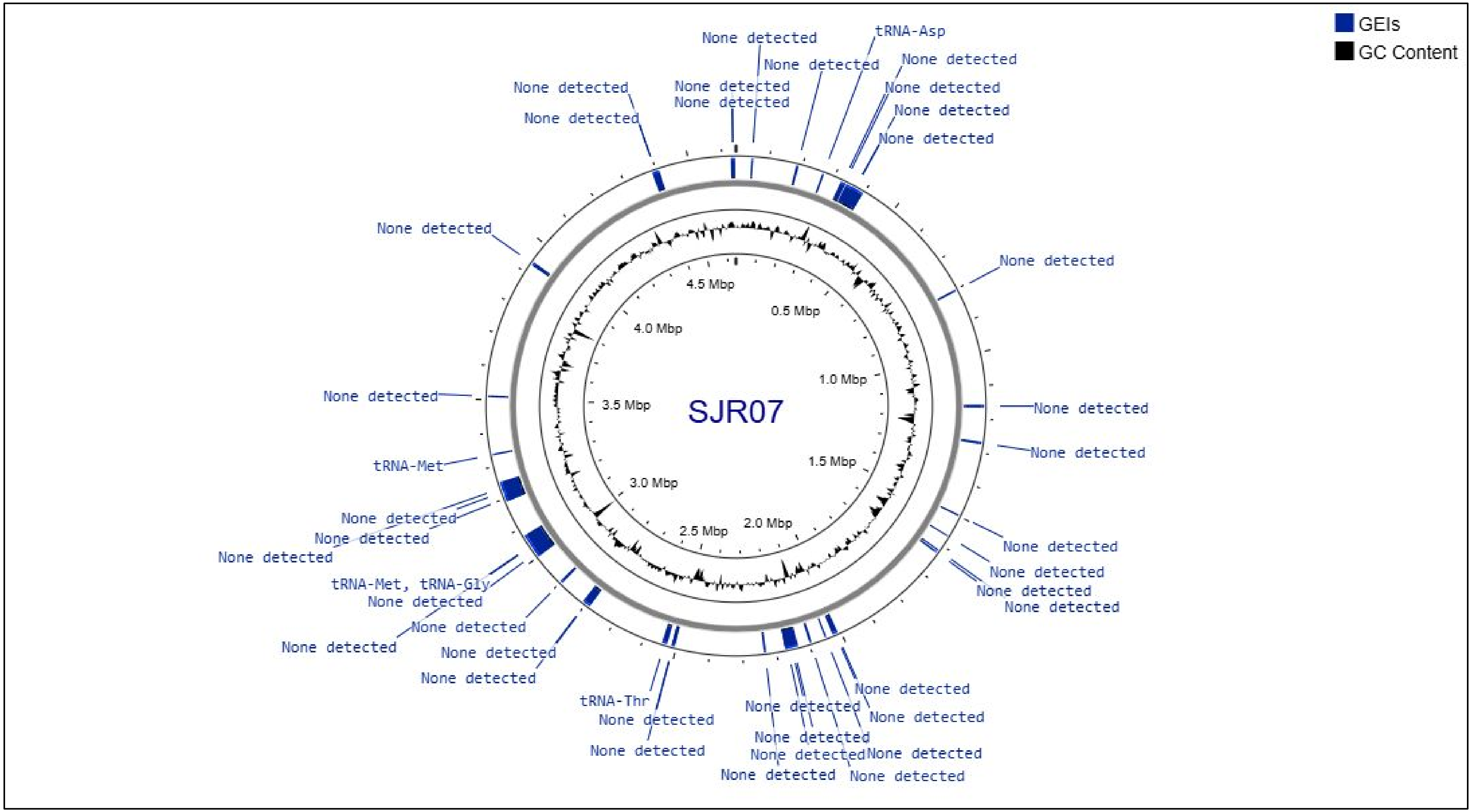

Figure S4. Genomic island boundaries and tRNA positions predicted in *E. coli* SJR07

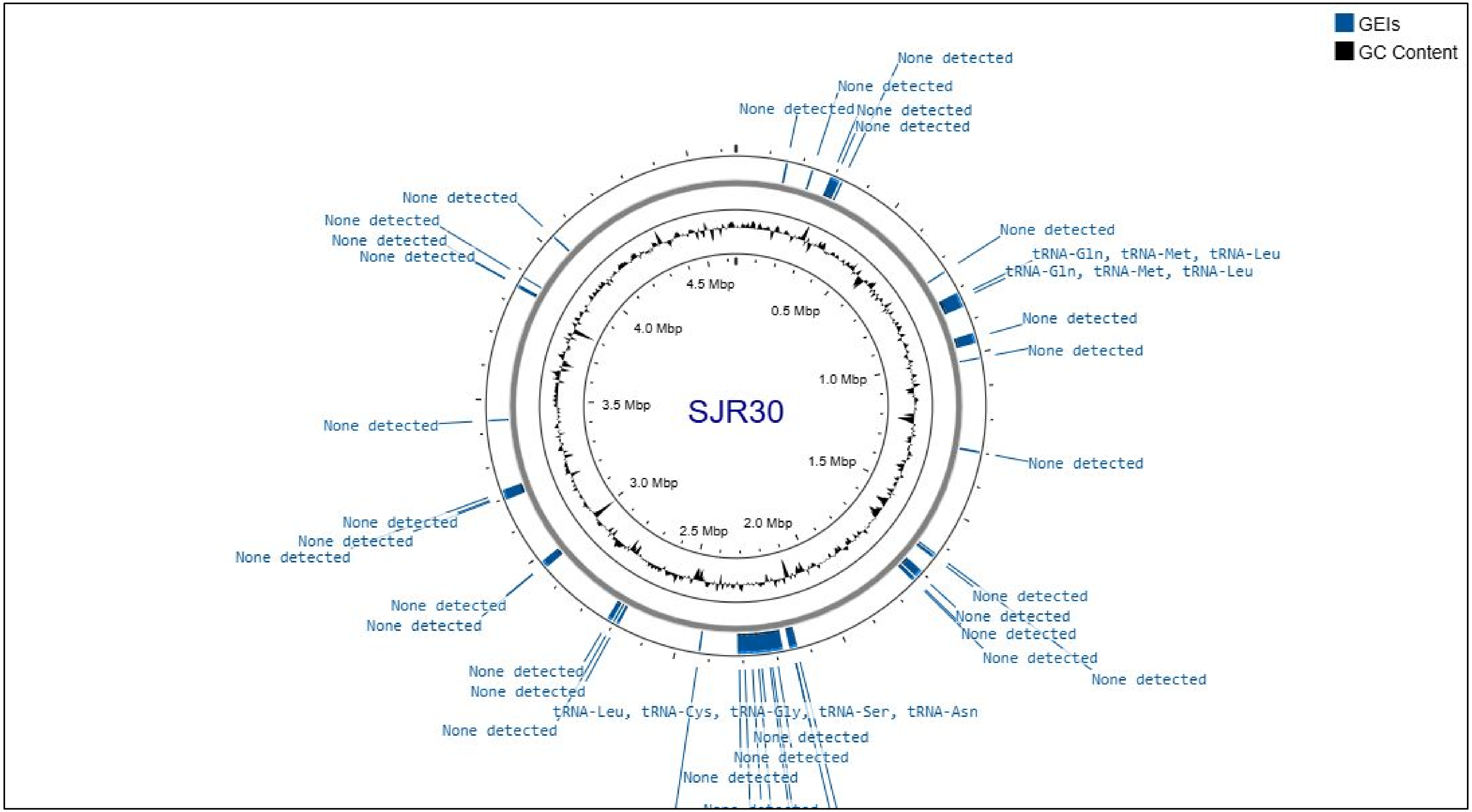

Figure S5. Genomic island boundaries and tRNA positions predicted in *E. coli* SJR30

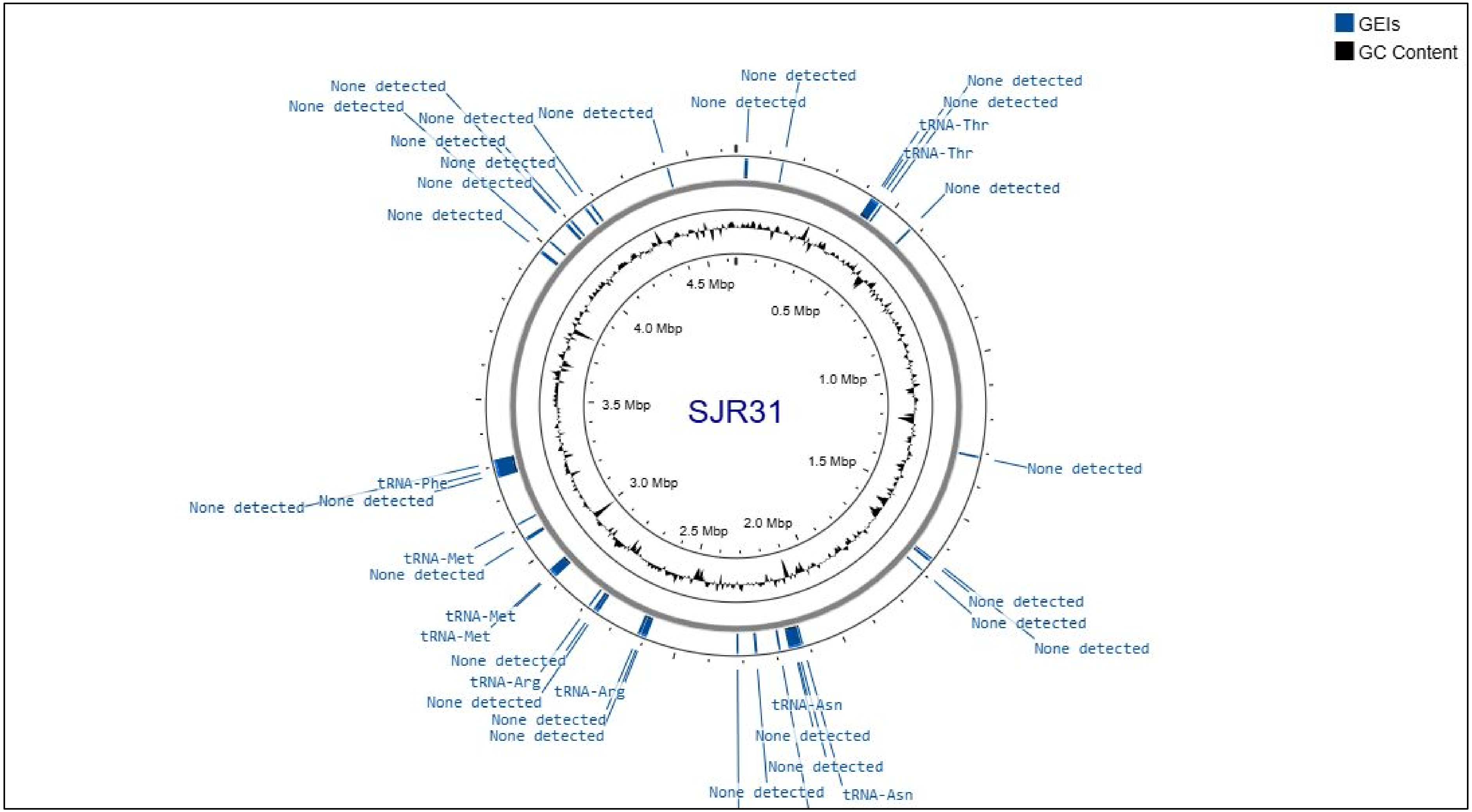
GEIs
GC Content
SJR31
4.5 Mbp
0.5 Mbp
4.0 Mbp
1.0 Mbp
3.5 Mbp
1.5 Mbp
3.0 Mbp
2.0 Mbp
2.5 Mbp
None detected
tRNA-Thr
tRNA-Thr
tRNA-Phe
tRNA-Met
tRNA-Met
tRNA-Met
tRNA-Arg
tRNA-Arg
tRNA-Asn
tRNA-Asn

Figure S6. Genomic island boundaries and tRNA positions predicted in *E. coli* SJR31

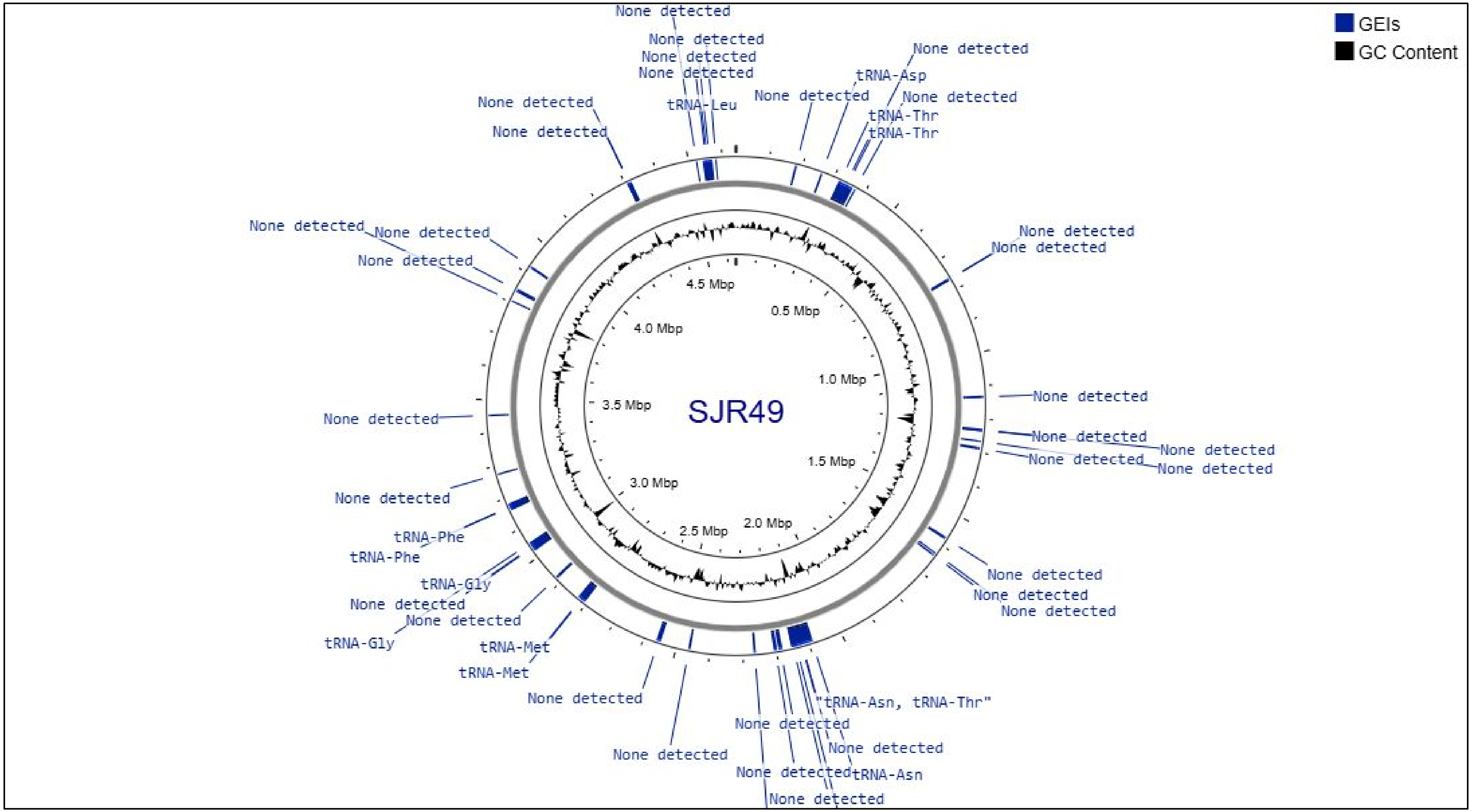
GEIs
GC Content
SJR49
4.5 Mbp
0.5 Mbp
4.0 Mbp
1.0 Mbp
3.5 Mbp
1.5 Mbp
3.0 Mbp
2.5 Mbp
2.0 Mbp
None detected
tRNA-Leu
tRNA-Asp
tRNA-Thr
tRNA-Thr
"tRNA-Asn, tRNA-Thr"
tRNA-Asn
tRNA-Met
tRNA-Met
tRNA-Gly
tRNA-Gly
tRNA-Phe
tRNA-Phe

Figure S7. Genomic island boundaries and tRNA positions predicted in *E. coli* SJR49

Table S1. Quality and assembly metrics of the analyzed genomes.

Name	RefSec	Completeness	Contamination	Contig_N50	Genome_Size	GC	Total_Contigs
L1LB	GCF_029414575.1	100	0.39	170705	4914842	0.51	108
L2BHI	GCF_029413755.1	100	0.02	115824	4970891	0.51	172
APEC S17	GCF_000340255.1	100	0.16	44480	4591470	0.51	187
SCU 204	GCF_000459135.1	100	0.95	5121232	5349412	0.5	6
EC958	GCF_000285655.3	100	0.97	5109767	5249449	0.51	3
PDT001089438	GCF_001763622.1	100	0.09	91210	4730255	0.51	156
PDT001090596	GCF_001764258.1	100	0.22	125578	5177688	0.51	196
UMN026	GCF_000026325.2	100	0.13	5202090	5202090	0.51	1
UTI89	GCF_000013265.1	100	0.83	5065741	5179971	0.51	2
CFT073	GCF_000007445.1	100	0.7	5231428	5231428	0.5	1
100516	GCF_003984305.1	100	0.04	88647	4946620	0.51	188
200144	GCA_014772715.1	100	1.13	75533	5096812	0.51	211
202973_aEPEC	GCF_001277735.1	100	0.15	191035	4742618	0.5	94
39_Esco_HA-DE	GCF_002442335.1	100	0.07	210315	4692670	0.51	44
3_2303	GCF_000194645.1	100	0	258736	4946233	0.5	35
500669	GCF_003981165.1	100	0.13	97104	4811407	0.5	159
602370_aEPEC	GCF_001277415.1	100	0.15	205023	4744889	0.5	85
681	GCF_000503335.1	100	0.04	73904	5051731	0.51	285
710469	GCF_003981355.1	100	0.11	88647	4878022	0.51	170
710499	GCF_003980495.1	100	0.11	81201	4860718	0.5	174
85B	GCF_000503415.1	100	0.11	53555	5071821	0.5	363

CFSAN061759	GCF_003399875.1	100	0.37	138086	4806092	0.51	110
CFT073	GCF_014262945.1	100	0.8	5242376	5242376	0.5	1
E2	GCF_001693555.1	100	4.81	74212	5081209	0.51	222
E2348_69	GCF_000026545.1	100	0.52	4965553	5069678	0.51	3
Ec_03	GCF_001893865.1	94.81	0.06	117526	4305255	0.5	94
F345	GCF_004767035.1	100	0.15	130428	4835025	0.51	115
F498	GCF_002319165.1	100	0.04	93085	5073312	0.51	203
FHI99	GCF_000951935.1	100	0.27	277391	5368397	0.51	42
FWSEC0049	GCF_005046385.1	100	0.26	77153	4987637	0.5	170
FWSEC0077	GCF_005045415.1	100	0.45	106405	5138266	0.51	108
FWSEC0400	GCF_005041485.1	100	0.14	111524	4863421	0.5	128
FWSEC0438	GCF_005041235.1	100	0.13	105845	4852452	0.5	122
G19	GCF_001575305.1	100	0.06	127574	4798782	0.51	87
H7	GCF_001900455.1	100	0.2	4807161	4807161	0.51	1
HVH164	GCF_000458495.1	100	0.13	3414105	4684494	0.51	8
MOD1-EC5677	GCF_002226905.1	100	0.04	90140	4879237	0.5	187
MOD1_EC5673	GCF_002227115.1	100	0.03	88572	4901007	0.5	156
MOD1_EC5691	GCF_002226695.1	100	0.13	78447	4622858	0.51	181
MOD1_EC5813	GCF_002474495.1	100	0.04	124554	4681635	0.51	134
MOD1_EC5862	GCF_002548165.1	100	0.06	139701	4908243	0.51	173
MOD1_EC5918	GCF_002534875.1	100	0.07	234633	4935221	0.51	83
MOD1_EC6478	GCF_002485665.1	100	0.41	99225	4736043	0.51	139
MOD1_EC6587	GCF_002469285.1	100	0.11	53518	5321249	0.51	384
MOD1_EC667	GCF_002466925.1	100	0.45	130397	5134412	0.51	192
P1016	GCA_014772715.1	100	1.53	118406	5237570	0.51	127
P1017	GCA_014752645.1	100	3.84	197925	5399312	0.51	118
P1035	GCA_014772675.1	100	2.93	176493	5100489	0.51	186
P4001	GCA_014753365.1	100	3.47	159607	5099852	0.51	133
P6034	GCA_014762005.1	100	4.15	158248	5302871	0.51	89

P6088	GCA_014751925.1	100	1.23	130475	5077272	0.51	126
P6136	GCA_014753465.1	100	4.15	203986	5263698	0.51	88
P6175	GCA_014759885.1	100	6.26	132355	5257137	0.51	181
P6211	GCA_014759885.1	100	3.69	154083	5215202	0.51	128
P6343	GCA_014759965.1	100	2.6	107998	5331560	0.51	157
P6374	GCA_014753125.1	100	3.82	138706	5291150	0.51	144
P6376	GCA_014751855.1	100	3.26	200523	4859680	0.51	76
SC352	GCF_003358245.1	100	0.01	111709	4962626	0.5	214
SC356	GCF_003358185.1	100	0.04	125573	4892010	0.5	183
STEC2708	GCF_001607685.1	100	0.06	101127	4793305	0.51	143
TW15901	GCF_000304075.2	100	0.05	119409	4897883	0.5	206
UMEA_3180_1	GCF_000460635.1	100	0.48	3256642	5156358	0.5	17
UMN026	GCF_000026325.1	100	0.37	5202090	5358200	0.51	3
UPEC129	GCF_017356665.1	100	0.74	5078748	5133980	0.51	3
UPEC132	GCF_007833875.1	100	0.96	5234468	5452963	0.51	3
UPEC245U_190328	GCF_023376095.1	100	0.29	5016451	5301911	0.51	7
UPEC455U_190220	GCF_019443605.1	100	0.72	5003672	5192928	0.51	6
UPEC774U_190308	GCF_019443685.1	100	0.81	4993321	5107728	0.51	5
UPEC_26-1	GCF_001693315.1	100	1.28	5178720	5329753	0.51	2
UPEC_U013	GCF_014522245.1	100	1.21	5203972	5203972	0.5	1
URMC_18	GCF_004568035.1	100	0.82	150179	4951002	0.51	86
VL2874	GCF_001441335.1	100	0.31	88833	4784284	0.51	126
Win2013_WWKa_OUT_18	GCF_003144805.1	100	0.06	88084	4766245	0.51	113
blood_10_1009	GCF_000779005.1	100	1.29	196851	4886174	0.51	90
blood_90187	GCF_000779155.1	100	0.05	223267	5044767	0.51	100
k12	GCF_000005845.2	100	0.13	4641652	4641652	0.51	1
nissle_1917	GCF_000714595.1	100	0.94	5055412	5064099	0.51	3
upec_127	GCF_000780965.1	100	0.23	119078	5139105	0.51	151

upec_173	GCF_000779955.1	100	0.4	152940	4873971	0.51	86
upec_282	GCF_000777535.1	100	0.08	162948	4693285	0.51	66
upec_83	GCF_000776355.1	100	0.05	105276	4892635	0.51	123

For each genome, the following data are shown: identifier (*Name*), reference accession (*RefSec*), completeness (%), contamination (%), N50 value of contigs (*Contig_N50*), total genome size (*Genome_Size*), GC content (*GC_Content*), and total number of contigs (*Total_Contigs*). These data were used to assess genome assembly quality and to select representative genomes for downstream analyses.

Table S2. UPEC isolates plasmid typing

Plasmid	Size (bp)	Rep_type(s)	Predicted_mobility	Mash_nearest_neighbor	Mash_neighbor_identification	Predicted_host_range_overall_rank	Predicted_host_range_overall_name
E_coli_SJR07_AA324	113491	IncFIA,IncFIA,IncFIC,IncFIA,IncFII,IncFIB	conjugative	LO017736	Escherichia coli	order	Enterobacterales
E_coli_SJR07_AB744	35567	-	mobilizable	CP010194	Escherichia coli	genus	Escherichia
E_coli_SJR07_AB595	6904	rep_cluster_2335	mobilizable	KT988306	Shigella sonnei	multi-phylla	Actinomycetota,Bacillota,Pseudomonadota

E_coli_SJR07_AA162	4696	ColRNAI_rep_cluster_1857,rep_cluster_2370	mobilizable	CP011433	Salmonella enterica Typhimurium	family	Enterobacteriaceae
E_coli_SJR07_AD481	4176	rep_cluster_2350,rep_cluster_2350	mobilizable	NC_014543	Escherichia coli O26:H11 str. 11368	genus	Escherichia
E_coli_SJR07_AA163	3408	-	mobilizable	CP021100	Escherichia coli	family	Enterobacteriaceae
E_coli_SJR07_AB687	1860	Col(MG828)	non-mobilizable	CP023506	Morganella morganii	order	Enterobacterales
E_coli_SJR30_AA747	111498	IncFIB	non-mobilizable	CP017847	Escherichia coli	order	Enterobacterales
E_coli_SJR31_novel	60111	IncQ1	non-mobilizable	CP026578	Escherichia coli	phylum	Pseudomonadota
E_coli_SJR31_AA174	77548	IncFIB,IncFII	conjugative	CP023361	Escherichia coli	order	Enterobacterales
E_coli_SJR49_AA315	61188	IncFIA,IncFIC	conjugative	CP013026	Escherichia coli	family	Enterobacteriaceae

E_coli_SJR49_AD094	5733	-	non-mobilizable	CP015133	Klebsiella pneumoniae	genus	Klebsiella
E_coli_SJR49_AA579	4110	-	mobilizable	CP019648	Salmonella enterica Typhimurium	family	Enterobacteriaceae

Table S3. Genomic island boundaries and tRNA coordinates in *E. coli* SJR07

Feature	Start	End	Strand	Integration_site
GI_1	45606	52030	+	None detected
GI_2	181263	188963	+	None detected
GI_3	262607	268766	+	tRNA-Asp
GI_4	322822	335288	+	None detected
GI_5	331800	338739	+	None detected
GI_6	340636	398025	+	None detected
GI_7	359435	381364	+	None detected
GI_8	799200	805545	+	None detected
GI_9	1156263	1165581	+	None detected
GI_10	1266873	1275291	+	None detected
GI_11	1495341	1500778	+	None detected
GI_12	1565291	1569498	+	None detected
GI_13	1615757	1620083	+	None detected
GI_14	1622944	1628566	+	None detected
GI_15	2008811	2025488	+	None detected
GI_16	2011180	2015269	+	None detected

GI_17	2044975	2050113	+	None detected
GI_18	2091293	2098903	+	None detected
GI_19	2133569	2173056	+	None detected
GI_20	2140117	2156079	+	None detected
GI_21	2158109	2173056	+	None detected
GI_22	2230762	2237808	+	None detected
GI_23	2504581	2514939	+	None detected
GI_24	2507443	2514939	+	None detected
GI_25	2528475	2544568	+	tRNA-Thr
GI_26	2786101	2812042	+	None detected
GI_27	2798894	2803494	+	None detected
GI_28	2893587	2902237	+	None detected
GI_29	2999059	3075164	+	tRNA-Met, tRNA-Gly
GI_30	3002184	3023853	+	None detected
GI_31	3036202	3041538	+	None detected
GI_32	3189268	3250758	+	None detected
GI_33	3189268	3209433	+	None detected
GI_34	3222308	3241621	+	None detected
GI_35	3332450	3337935	+	tRNA-Met
GI_36	3507658	3512925	+	None detected
GI_37	3927075	3938424	+	None detected
GI_38	4386692	4408986	+	None detected
GI_39	4390913	4403682	+	None detected
GI_40	4626467	4638032	+	None detected
GI_41	4629059	4638032	+	None detected
GI_42	4639179	4647598	+	None detected
GI_43	4695053	4948550	+	tRNA-Arg, tRNA-Glu, tRNA-Ile, tRNA-Ala
GI_44	4744069	4762924	+	None detected

GI_45	4806759	4867281	+	tRNA-Ile, tRNA-Ala
GI_46	4873845	4918280	+	tRNA-Glu
GI_47	4928506	4953318	+	None detected

Table S4. Genomic island boundaries and tRNA coordinates in *E. coli* SJR30

Feature	Start	End	Strand	Integration_site
GI_1	149436	155964	+	None detected
GI_2	228915	235076	+	None detected
GI_3	289760	318348	+	None detected
GI_4	289760	294864	+	None detected
GI_5	323838	329329	+	None detected
GI_6	736017	740984	+	None detected
GI_7	810092	855951	+	tRNA-Gln, tRNA-Met, tRNA-Leu
GI_8	815415	830753	+	tRNA-Gln, tRNA-Met, tRNA-Leu
GI_9	939146	970884	+	None detected
GI_10	1014218	1019033	+	None detected
GI_11	1295615	1302722	+	None detected
GI_12	1629920	1634246	+	None detected
GI_13	1637107	1646186	+	None detected
GI_14	1696277	1720706	+	None detected
GI_15	1724941	1733776	+	None detected
GI_16	1731394	1736664	+	None detected
GI_17	2135956	2143565	+	None detected
GI_18	2141489	2160437	+	None detected
GI_19	2148529	2155685	+	None detected
GI_20	2179629	2315712	+	tRNA-Leu, tRNA-Cys, tRNA-Gly, tRNA-Ser, tRNA-Asn

GI_21	2195792	2208223	+	None detected
GI_22	2216040	2220067	+	None detected
GI_23	2221714	2227243	+	None detected
GI_24	2254635	2259195	+	tRNA-Asn
GI_25	2269669	2278689	+	None detected
GI_26	2290929	2299599	+	None detected
GI_27	2306443	2315712	+	None detected
GI_28	2427715	2433549	+	None detected
GI_29	2685084	2693739	+	None detected
GI_30	2698802	2704249	+	None detected
GI_31	2708816	2723467	+	None detected
GI_32	2959496	2982713	+	None detected
GI_33	2966231	2973896	+	None detected
GI_34	3194515	3226032	+	None detected
GI_35	3197640	3215804	+	None detected
GI_36	3218165	3222278	+	None detected
GI_37	3434462	3439938	+	None detected
GI_38	3847906	3858320	+	None detected
GI_39	3852438	3857031	+	None detected
GI_40	3881901	3885923	+	None detected
GI_41	4031222	4037099	+	None detected
GI_42	4705238	4709420	+	tRNA-Phe
GI_43	4726798	4757065	+	tRNA-Gly
GI_44	4734899	4740868	+	tRNA-Gly
GI_45	4761092	4771689	+	None detected
GI_46	4918959	4928154	+	tRNA-Thr, tRNA-Ile, tRNA-Ala
GI_47	4934001	4943081	+	None detected

Table S5. Genomic island boundaries and tRNA coordinates in *E. coli* SJR31

Feature	Start	End	Strand	Integration_site
GI_1	25923	36409	+	None detected
GI_2	139171	143583	+	None detected
GI_3	426498	451753	+	tRNA-Thr
GI_4	426498	439335	+	tRNA-Thr
GI_5	445089	453929	+	None detected
GI_6	460767	466259	+	None detected
GI_7	571448	578228	+	None detected
GI_8	1310877	1317375	+	None detected
GI_9	1647268	1651593	+	None detected
GI_10	1654459	1661825	+	None detected
GI_11	1692035	1696747	+	None detected
GI_12	2116744	2121818	+	tRNA-Asn
GI_13	2123273	2162689	+	tRNA-Asn
GI_14	2131689	2139837	+	None detected
GI_15	2141965	2152160	+	None detected
GI_16	2185981	2192852	+	None detected
GI_17	2256606	2264665	+	None detected
GI_18	2313263	2319103	+	None detected
GI_19	2593881	2612174	+	tRNA-Arg
GI_20	2606392	2610619	+	None detected
GI_21	2613628	2623176	+	None detected
GI_22	2755733	2764436	+	None detected
GI_23	2764732	2773461	+	None detected
GI_24	2785692	2791242	+	tRNA-Arg
GI_25	2920251	2949361	+	tRNA-Met

GI_26	2936144	2941224	+	tRNA-Met
GI_27	3057278	3068236	+	None detected
GI_28	3110312	3115541	+	tRNA-Met
GI_29	3263922	3319079	+	tRNA-Phe
GI_30	3269487	3283761	+	None detected
GI_31	3305385	3319079	+	None detected
GI_32	3970872	3981814	+	None detected
GI_33	4013097	4019247	+	None detected
GI_34	4082978	4089323	+	None detected
GI_35	4086706	4092583	+	None detected
GI_36	4100834	4107817	+	None detected
GI_37	4157510	4165199	+	None detected
GI_38	4177925	4184780	+	None detected
GI_39	4431620	4437128	+	None detected
GI_40	4808209	4812486	+	None detected
GI_41	4835878	4876863	+	tRNA-Leu
GI_42	4850296	4857452	+	None detected
GI_43	4868351	4876863	+	None detected
GI_44	4900150	4947307	+	None detected
GI_45	4908351	4966803	+	None detected
GI_46	4975178	5045597	+	None detected
GI_47	4975688	4988513	+	None detected
GI_48	5030887	5045245	+	None detected

Table S6. Genomic island boundaries and tRNA coordinates in *E. coli* SJR49

feature	start	stop	strand	Integration_site
GI_1	177632	184118	+	None detected

GI_2	257892	264051	+	tRNA-Asp
GI_3	316208	363377	+	tRNA-Thr
GI_4	317658	324861	+	tRNA-Thr
GI_5	342045	347927	+	None detected
GI_6	368774	373685	+	None detected
GI_7	759248	768298	+	None detected
GI_8	761728	767641	+	None detected
GI_9	1129253	1137033	+	None detected
GI_10	1227896	1236827	+	None detected
GI_11	1230431	1237242	+	None detected
GI_12	1263779	1269209	+	None detected
GI_13	1283665	1292083	+	None detected
GI_14	1571891	1580664	+	None detected
GI_15	1628035	1632361	+	None detected
GI_16	1635222	1640841	+	None detected
GI_17	2086787	2094398	+	tRNA-Asn
GI_18	2092322	2153517	+	tRNA-Asn, tRNA-Thr
GI_19	2115138	2126875	+	None detected
GI_20	2135523	2142427	+	tRNA-Thr, tRNA-Asn
GI_21	2143853	2153517	+	None detected
GI_22	2180646	2193534	+	None detected
GI_23	2197045	2207430	+	None detected
GI_24	2261102	2267943	+	None detected
GI_25	2457798	2465046	+	None detected
GI_26	2548695	2563346	+	None detected
GI_27	2805590	2831532	+	tRNA-Met
GI_28	2818384	2822984	+	tRNA-Met
GI_29	2913262	2921912	+	None detected
GI_30	3019837	3049590	+	tRNA-Gly

GI_31	3022962	3042131	+	None detected
GI_32	3044482	3048528	+	tRNA-Gly
GI_33	3160320	3181586	+	tRNA-Phe
GI_34	3160320	3180598	+	tRNA-Phe
GI_35	3271673	3277158	+	None detected
GI_36	3450325	3455592	+	None detected
GI_37	3802581	3807579	+	None detected
GI_38	3834811	3846160	+	None detected
GI_39	3917100	3924885	+	None detected
GI_40	4305112	4319597	+	None detected
GI_41	4305112	4317255	+	None detected
GI_42	4521416	4525782	+	None detected
GI_43	4541561	4571465	+	tRNA-Leu
GI_44	4548869	4555885	+	None detected
GI_45	4559301	4566245	+	None detected
GI_46	4579600	4584043	+	None detected
GI_47	4671342	4806924	+	None detected
GI_48	4677184	4692230	+	None detected
GI_49	4696132	4708366	+	None detected
GI_50	4731412	4737017	+	None detected
GI_51	4740023	4744851	+	None detected
GI_52	4778610	4792066	+	None detected
GI_53	4800344	4806924	+	None detected

Newick file for MLST-based tree

(MOD1_EC5673:0.00000100000050002909,((E2:0.00000100000050002909,(ST10_L2BHI_Peru_Global_2019:0.00000100000050002909,ST10_562_34097_B
razil_animal_1998:0.00000100000050002909):0.00000100000050002909[3]):0.00000100000050002909[0],((602370_aEPEC:0.00000100000050002909,CFS
AN061759:0.00000100000050002909):0.00000100000050002909[4],(((FWSEC0077:0.00000100000050002909,(((681:0.00000100000050002909,((((((MOD
1_EC5813:0.00000100000050002909,MOD1-
EC5677:0.00000100000050002909):0.00000100000050002909[4],FWSEC0049:0.00000100000050002909):0.00000100000050002909[0],H7:0.00000100000
050002909):0.00000100000050002909[0],MOD1_EC667:0.00000100000050002909):0.00000100000050002909[0],STEC2708:0.00000100000050002909):0.
00000100000050002909[0],MOD1_EC6478:0.00000100000050002909):0.00000100000050002909[0]):0.00000100000050002909[0],(ST10_L1LB_Peru_Glo
bal_2019:0.00000100000050002909,(Ec_03:0.00000100000050002909,(((upec_83:0.00000100000050002909,upec_173:0.00000100000050002909):0.0000
0100000050002909[3],E_coli_SJR07:0.00000100000050002909):0.00000100000050002909[0],(SC352:0.00000100000050002909,MOD1_EC5862:0.000001
00000050002909):0.00000100000050002909[2]):0.00000100000050002909[0]):0.00000100000050002909[0]):0.00000100000050002909[0]):0.000001000
00050002909[0],(HVH164:0.00000100000050002909,(P6088:0.00033881513353722204,(((MOD1-EC1641:0.00437988348507713579,(MOD1-
EC4358:0.00552952193427788240,Shigella_flexneri_FC1170:0.00571081184623769764):0.00187592462284885686[40]):0.00155268164365604078[34],((((
E_coli_SJR31:0.00000100000050002909,ST354_PDT001090596_1_Brazil_2019:0.00000100000050002909):0.00623994437874014242[100],WB61:0.003534
83507342716285):0.00495501281833759296[90],((HVH_65:0.00603894038549292189,(((P1016:0.00000100000050002909,P6343:0.000001000000500029
09):0.00000100000050002909[32],FVEC1412:0.00000100000050002909):0.00000100000050002909[55],(ST69_UMN026_USA_Global_2022:0.0000010000
0050002909,UMN026:0.00000100000050002909):0.00033921462263391254[76]):0.00488194808252238096[99]):0.00363433466884500100[63],MOD1-
EC5921:0.00433888674694696708):0.00351171383041495562[52]):0.00428222471950086628[59],((MOD1-
EC6621:0.00725710034205893570,(((((P6374:0.00000100000050002909,P6211:0.00000100000050002909):0.00000100000050002909[20],ST131_EC958_A
ustralia_global_2014:0.00000100000050002909):0.00000100000050002909[17],(BIDMC_63:0.00000100000050002909,Eco889:0.00000100000050002909)
:0.00000100000050002909[43]):0.00383014530055242764[98],(((P6376:0.00033920977491513340,((ST95_UTI89_USA_Global_2006:0.0000010000005000
2909,P6034:0.00000100000050002909):0.00000100000050002909[55],(P6136:0.00000100000050002909,UPEC129:0.00000100000050002909):0.0000010
0000050002909[74]):0.00000100000050002909[46]):0.00211550796923563877[99],((UPEC132:0.00223524524337584104,P1035:0.0022482235878281977
2):0.00167710620435647319[87],((ST73_CFT073_USA_Global_2002:0.00000100000050002909,((CFT073:0.00000100000050002909,(P4001:0.00000100000
050002909,P6175:0.00000100000050002909):0.00000100000050002909[14]):0.00000100000050002909[6],GSK25213:0.00000100000050002909):0.00000
100000050002909[14]):0.00000100000050002909[74],(UPEC_U013:0.00000100000050002909,(nissle_1917:0.00000100000050002909,UPEC_26-
1:0.00000100000050002909):0.00000100000050002909[30]):0.00000100000050002909[69]):0.00160935945156194808[96]):0.00118000792994678522[5
1]):0.00170591171994071214[76],(((UPEC455U_190220:0.00000100000050002909,UPEC774U_190308:0.00000100000050002909):0.00000100000050002
909[33],ST1193_SCU_204_Emergent_USA_2022:0.00000100000050002909):0.00034493254905578505[84],P1017:0.00033829113290253718):0.00313935
868426562428[99]):0.00063480822301956511[29]):0.00071168851240223212[42],E2348_69:0.00278191479018617422):0.00343754386461840155[78]):0

.00503000151734189771[81],E_fergusonii:0.09567709860029531554):0.00051497328082839695[18]):0.00067567889198541129[15]):0.003101280722754
57981[32],((MOD1-EC3802:0.00136622609235582623,(E_coli_SJR30:0.00102883511682250791,(UPEC245U_190328:0.00171832547390379721,2014C-
4638:0.00102940720306169613):0.00137124511460325234[44]):0.00034782739913728398[29]):0.00098701151855471944[34],Shigella_sonnei_CDPH_C9
6:0.00352350084768896605):0.00172507205024815657[78]):0.00914288390414820171[85]):0.00000100000050002909[18]):0.00000100000050002909[0]
):0.00000100000050002909[0]):0.00000100000050002909[0],((Win2013_WWKa_OUT_18:0.00033900979214249161,(ST167_PDT001089438_1_Brazil_201
9:0.00000100000050002909,E_coli_SJR49:0.00000100000050002909):0.00136665867217847584[97]):0.00000100000050002909[9],(((((((SC356:0.000001
00000050002909,MOD1_EC6587:0.00000100000050002909):0.00000100000050002909[4],((((((85B:0.00000100000050002909,blood_90187:0.000001000
00050002909):0.00000100000050002909[4],(upec_127:0.00000100000050002909,k12:0.00000100000050002909):0.00000100000050002909[3]):0.00000
100000050002909[0],((blood_10_1009:0.00000100000050002909,(200144:0.00000100000050002909,710469:0.00000100000050002909):0.00000100000
050002909[4]):0.00000100000050002909[0],500669:0.00000100000050002909):0.00000100000050002909[0]):0.00000100000050002909[0],TW15901:0.0
0000100000050002909):0.00000100000050002909[0],710499:0.00000100000050002909):0.00000100000050002909[0],F345:0.00000100000050002909):
0.00000100000050002909[0],((104:0.00000100000050002909,MOD1_EC5691:0.00000100000050002909):0.00000100000050002909[1],F498:0.000001000
00050002909):0.00000100000050002909[0]):0.00000100000050002909[0]):0.00000100000050002909[0],FWSEC0400:0.00000100000050002909):0.00000
100000050002909[0],FWSEC0438:0.00000100000050002909):0.00000100000050002909[0],VL2874:0.00000100000050002909):0.00000100000050002909
[0],MOD1_EC5918:0.00000100000050002909):0.00000100000050002909[0],UMEA_3180_1:0.00000100000050002909):0.00000100000050002909[0],(ST1
0_S17_APEC_Brazil_animal:0.00000100000050002909,FHI99:0.00000100000050002909):0.00000100000050002909[2]):0.00000100000050002909[12]):0.0
0000100000050002909[4]):0.00000100000050002909[0],(100516:0.00000100000050002909,((202973_aEPEC:0.00000100000050002909,((3_2303:0.00000
100000050002909,39_Esco_HA-
DE:0.00000100000050002909):0.00000100000050002909[5],upec_282:0.00000100000050002909):0.00000100000050002909[0]):0.000001000000500029
09[0],G19:0.00000100000050002909):0.00000100000050002909[0]):0.00000100000050002909[0]):0.00000100000050002909[0]):0.000001000000500029
09[0]):0.00000100000050002909[1],URMC_18:0.00000100000050002909);

Newick format for Coregenome-based tree

(((E_coli_SJR49:0.00067101763724286426,(E_coli_SJR07:0.00034845520231103968,ST167_PDT001089438_1:0.00036980383182953731):0.001201935090
62157134[100]):0.00052466895553441510[100],(ST10_L1LB:0.00000100000050002909,ST10_L2BHI:0.00000100000050002909):0.00126890445734801445
[100]):0.00033330209961881044[100],(ST10_562:0.00200337144353397549,((Shigella_flexneri_FC1170:0.00934751771738391406,((E_coli_SJR30:0.00353
371464366635879,MOD1-EC3802:0.00352130530223121159):0.00149973432173333351[100],2014C-
4638:0.00343331190396348965):0.00319808062916928466[100]):0.00207065505208977327[100],(((ST69_UMN026:0.00843966676862839549,HVH_65:0.
00855791892895141348):0.00262537655212693056[100],MOD1-

EC5921:0.00956537343709101644):0.00374889605772759250[100],(E_fergusonii:0.16537827438523547419,((ST354_PDT001090596_1:0.0005346072201
3671353,E_coli_SJR31:0.00064800748235305445):0.01179118657974908939[100],(MOD1-
EC6621:0.01002351460012812674,(ST131_EC958:0.00627118001744381148,((ST95_UTI89:0.00435971070640233781,ST73_CFT073:0.0046821813129157
5114):0.00179105176204928489[100],ST1193_SCU204:0.00589722200036466460):0.00206791917833453300[100]):0.00947024518070426966[100]):0.00
432983097950828157[100]):0.00147436496318786598[100]):0.00174866195167406994[100]):0.00857952003654886483[100]):0.00624591297914127170
[100]):0.00080382170902289815[100],ST10_S17:0.00119165260562285294);
